# DNA microarray of global transcription factor mutant reveals membrane-related proteins involved in *n*-butanol tolerance in *Escherichia coli*

**DOI:** 10.1186/s13068-016-0527-9

**Published:** 2016-06-01

**Authors:** Hai-Ming Si, Fa Zhang, An-Ning Wu, Rui-Zhi Han, Guo-Chao Xu, Ye Ni

**Affiliations:** The Key Laboratory of Industrial Biotechnology, Ministry of Education, School of Biotechnology, Jiangnan University, Wuxi, 214122 Jiangsu China

**Keywords:** Butanol tolerance, DNA microarrays, Membrane-related proteins, Global transcription machinery engineering, *Escherichia coli*

## Abstract

**Background:**

*Escherichia coli* has been explored as a platform host strain for biofuels production such as butanol. However, the severe toxicity of butanol is considered to be one major limitation for butanol production from *E. coli*. The goal of this study is therefore to construct butanol-tolerant *E. coli* strains and clarify the tolerance mechanisms.

**Results:**

A recombinant *E. coli* strain harboring σ^70^ mutation capable of tolerating 2 % (v/v) butanol was isolated by the global transcription machinery engineering (gTME) approach. DNA microarrays were employed to assess the transcriptome profile of butanol-tolerant strain B8. Compared with the wild-type strain, 329 differentially expressed genes (197 up-regulated and 132 down-regulated) (*p* < 0.05; FC ≥ 2) were identified. These genes are involved in carbohydrate metabolism, energy metabolism, two-component signal transduction system, oxidative stress response, lipid and cell envelope biogenesis and efflux pump.

**Conclusions:**

Several membrane-related proteins were proved to be involved in butanol tolerance of *E. coli*. Two down-regulated genes, *yibT* and *yghW*, were identified to be capable of affecting butanol tolerance by regulating membrane fatty acid composition. Another down-regulated gene *ybjC* encodes a predicted inner membrane protein. In addition, a number of up-regulated genes, such as *gcl* and *glcF*, contribute to supplement metabolic intermediates for glyoxylate and TCA cycles to enhance energy supply. Our results could serve as a practical strategy for the construction of platform *E. coli* strains as biofuel producer.

**Electronic supplementary material:**

The online version of this article (doi:10.1186/s13068-016-0527-9) contains supplementary material, which is available to authorized users.

## Background

Concerns on global energy crisis and environmental problems have prompted the development of renewable biofuels as potential alternatives for replacing traditional fossil fuels. Among biofuels, butanol has attracted much attention due to its higher energy density, miscibility with gasoline and lower corrosivity [1]. *Escherichia coli*, as an important platform microorganism, has been widely engineered as an alternative host for the production of various biofuels due to its advantages of fast growth and easy genetic manipulation [2, 3]. However, most biofuels are toxic to *E. coli*, which barely tolerate organic solvents with Log*P* values lower than 3.4–3.8 [4]. For example, cell growth of *E. coli* is completely inhibited in the presence of 1 % (v/v) *n*-butanol [5]. The poor butanol tolerance of *E. coli* has been a major limitation in the development of butanol-producing strains. Therefore, it is urgently necessary to improve the butanol tolerance of *E. coli*.

Most industrial biofuel-producing strains were obtained through solvent stress adaptation, genetic and metabolic engineering and traditional mutagenesis [6–9]. However, long-term adaptive evolution and traditional mutagenesis are often time-consuming. Significantly improved microbial tolerance requires a complex and multigenic phenotype. With recent development of direct mapping between the transcriptome and phenotype, strain improvement efforts have been focused on the manipulation of transcription factors. Global transcription machinery engineering (gTME) emerged as a promising strategy and has been widely used to evolve the desired phenotypes in recent years [10, 11]. Several transcription factors, including sigma factor, CRP, *Spt15*, H-NS and Hha, have been successfully engineered to improve organic solvent tolerance of various microbial strains [12–15]. σ^70^ is a subunit of RNA polymerase encoded by *rpoD*, which regulates over 1000 genes in *E. coli*. Studies showed that σ^70^ mutations could alter the promoter preferences of RNA polymerase and therefore affect transcriptome at a global level [16–18]. Our group previously isolated an *E. coli* harboring σ^70^ mutant C9, which could grow in the presence of 69 % (v/v) of cyclohexane [19].

Many solvent tolerance-related genes and their mechanisms have been reported in *E. coli* strains. MarA (encoded by *marA*) was confirmed to be a transcription factor capable of inducing the expression of mar-sox regulon genes [20, 21]. Disruption of *proV* and *marR* genes could increase the *n*-hexane tolerance of *E. coli* cells, possibly due to their function in regulation of osmotic pressure [22]. Rutherford and coworkers reported that n-butanol stress response genes are also involved in other stress responses, such as oxidative stress (*sodA, sodC and yqhD*), heat shock and cell envelope stress (*rpoE*, *clpB*, *htpG*, *cpxR* and *cpxP*) [23]. In our previous study, *E. coli* strain overexpressing *mmsB* (encoding 3-hydroxyisobutyrate dehydrogenase) exhibited high solvent tolerance by generating more energy to pump out intracellular organic solvent [24]. These studies demonstrate the complexity and diversity of solvent tolerance mechanisms in *E. coli*.

It has been reported that *E. coli* strain capable of growing in the presence of 1.2 % (v/v) butanol was obtained by engineering the global transcription factor cyclic AMP receptor protein [13]. Recently, n-butanol tolerance of *E. coli* was expanded to 2 % (v/v) by engineering an artificial transcription factor combining with controlling membrane-related functions [25]. In this work, we aimed to improve the butanol tolerance of *E. coli* by a feasible and efficient approach gTME and understand the mechanism between *rpoD* mutagenesis and the evolved phenotype. An *E. coli* strain carrying σ^70^ mutant capable of tolerating as high as 2 % (v/v) butanol was isolated, which is close to the highest butanol tolerance level of *E. coli* reported so far. DNA microarrays were employed to identify critical genes related to the n-butanol tolerance based on the transcriptome profile of mutant B8 compared with its wild type (WT). Several membrane-related genes (such as *yibT*, *yghW* and *ybjC*) were recognized to affect membrane fatty acid composition or function as a membrane protein. Other genes (such as *gcl* and *glcF*) involved in enhancing metabolic intermediates levels for glyoxylate and TCA cycles were also identified.

## Results and discussion

### Isolation of σ^70^ mutants

**Fig. 1:**
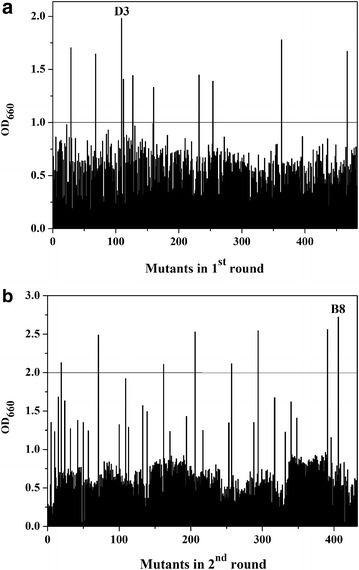
Screening for σ^70^ mutants with high butanol tolerance in the **a** first round and **b** second round. All mutant strains were cultured in 24-well plates at 37 °C for 8 h, and 0.5 % (v/v) n-butanol was added at 0.2 OD_660_

To obtain σ^70^ mutants with high n-butanol tolerance, random mutagenesis was performed to construct a *rpoD* mutant library of around 10^6^. In the first round of screening, 483 mutants were selected from the mutagenesis library on agar plate containing 0.5 % (v/v) butanol. They were further inoculated into 24-well plate under butanol pressure. Among them, ten mutants with OD_660_ of 1.3–2.0 were selected due to their advantageous growth under 0.5 % (v/v) butanol than other mutants (OD_660_ < 1) (Fig. 1a). Then these mutants were cultured in medium containing 0.5–1.5 % (v/v) n-butanol (0.1 % gradient increasing). One mutant exhibited the highest tolerance (0.76 OD_660_) at 1.2 % (v/v) n-butanol and was designated as D3, which was confirmed with two amino acid substitutions (I41L, P97Q).

**Fig. 2:**
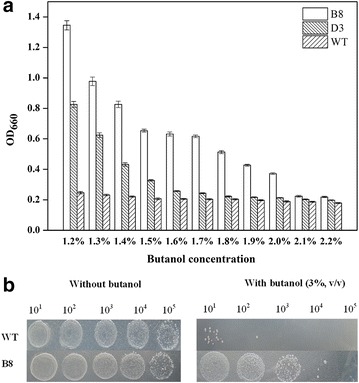
Assessment of butanol tolerance of σ^70^ mutants B8, D3 and WT. **a** The growth of B8, D3 and WT in the presence of different concentrations of n-butanol (1.2–2.2 %, v/v). All mutant strains were cultured in 24-well plates at 37 °C for 8 h. Different concentrations of n-butanol were added at 0.2 OD_660_. Three biological replicates were performed. **b** Butanol shock treatment of σ^70^ mutant B8 and WT. The strains viability was tested after treatíng without or with 3.0 % (v/v) butanol. Sequential tenfold dilution of cell cultures was spotted on LB agar and incubated at 37 °C overnight

To further improve the butanol tolerance, the second round of random mutagenesis was performed to construct a variant library using D3 as a template. In the second round of screening, eight mutants exhibited higher butanol tolerance (OD_660_ > 2.0) than others (Fig. 1b). In further screening under 0.5–1.5 % (v/v) butanol (0.1 % gradient increasing), one mutant B8 with three substitutions (I41L, E57D, P97Q) exhibited excellent growth in the presence of 1.2 % (v/v) butanol, reaching 1.432 OD_660_ compared with 0.778 of D3 and 0.247 of WT (Additional file 1: Figure S1). Then, mutants B8, D3 and WT were further cultured under higher n-butanol concentration (1.2–2.2 %) to assess their maximum butanol tolerance (Fig. 2a). The cell growth of WT was completely inhibited when butanol reached over 1.2 % (v/v), and mutant D3 could hardly grow in the presence of 1.6 % (v/v) butanol. Nevertheless, for mutant B8, OD_660_ reached 0.372 after 8 h incubation with 2.0 % (v/v) butanol, suggesting a much higher butanol tolerance than D3 and WT, while no cell growth was observed for B8 at over 2.0 % (v/v) n-butanol. Finally, B8 was selected as the best candidate for further study.

### Characterization of butanol-tolerant mutants

#### Solvent shock experiments

To further characterize the tolerance of B8, solvent shock experiments were performed in medium containing higher concentration of n-butanol. As shown in Fig. 2b, the growth of B8 and WT has no significant difference in the absence of butanol. After treatment with 3 % (v/v) butanol, WT showed growth only at tenfold dilution, while B8 could grow well at 10^3^-fold dilution on LB agar plate, indicating B8 has significantly higher butanol tolerance than WT.

#### Cell morphology

**Fig. 3:**
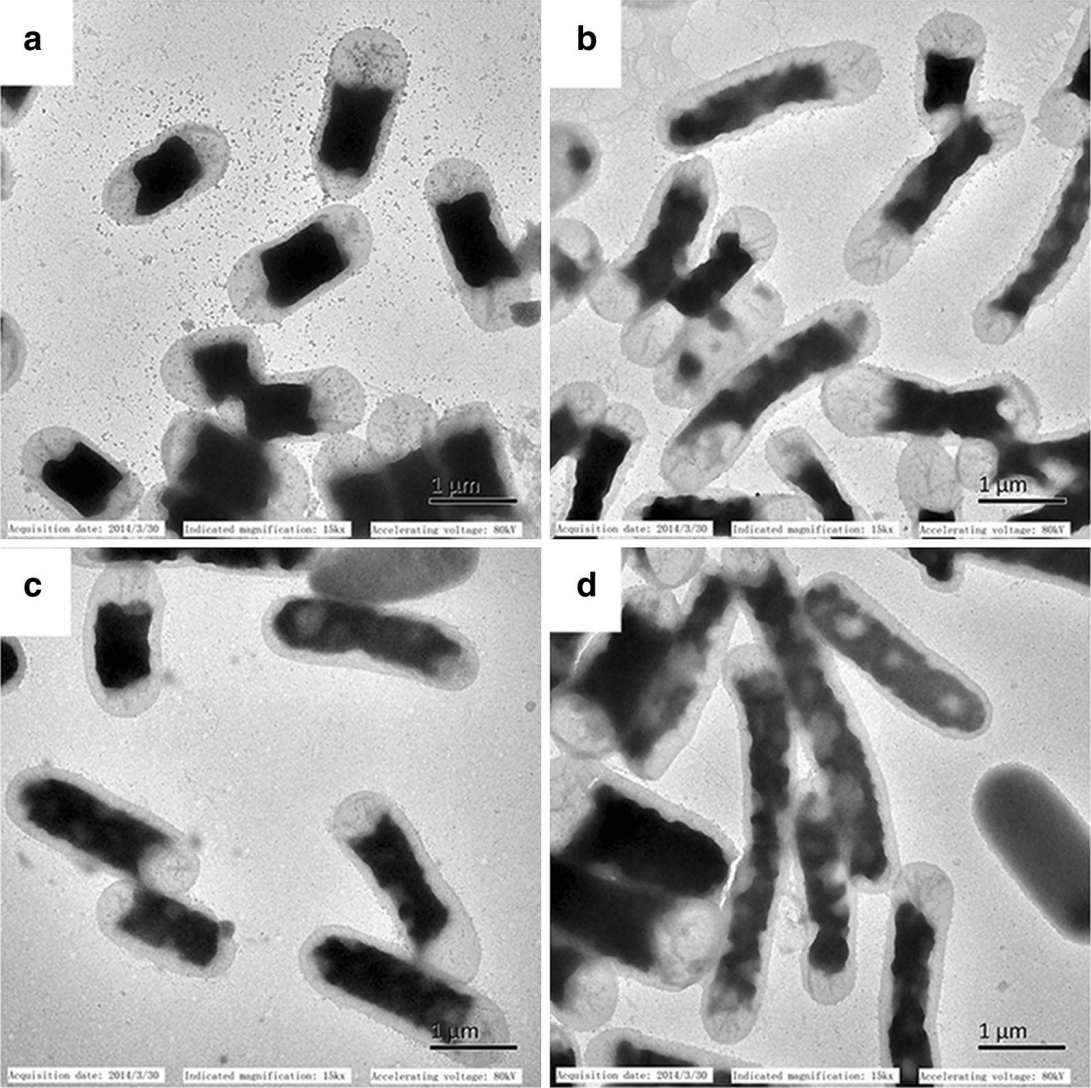
Transmission electron micrographs of σ^70^ mutant B8 and WT cells when cultured without or with 0.8 % (v/v) butanol (12,000× magnification). **a** WT without n-butanol. **b** WT with n-butanol. **c** B8 without n-butanol. **d** B8 with n-butanol

The cell morphology of microorganisms can adapt to the environmental changes [26]. Both WT and B8 grew in the absence or presence of butanol. In transmission electron microscope observation (Fig. 3), mutant B8 cells were significantly longer than WT cells after butanol treatment. In the absence of n-butanol, the cell size was (1.72 ± 0.17) × 0.73 μm for WT (Fig. 3a) and (1.89 ± 0.22) × 0.87 μm for B8 (Fig. 3c), respectively. After 0.8 % (v/v) n-butanol treatment, the cell size of WT and B8 shifted to (2.18 ± 0.25) × 0.53 μm (Fig. 3b) and (2.94 ± 0.39) × 0.52 μm, respectively (Fig. 3d), based on the measurement of around 100 cells. The changes in cell size were confirmed to be statistically significant (*p* < 0.05). Although the cell morphology was observed based on 100 cells per strain, the cell sizes could also be affected by reasons unrelated to the tolerance effects. Notably, B8 cells were longer than WT when subjected to n-butanol stress. A similar observation on the cell size of ethanol-adapted *Saccharomyces cerevisiae* has also been reported [27]. Neumann and coworkers reported that the microorganism cells of larger size could be more advantageous over the smaller ones under stress conditions, because the ratio of surface area to volume (S:V = 4πr^2^:4/3πr^3^) of larger cells is relatively lower than that of the smaller ones [28]. Similarly, B8 cells with smaller S:V value exhibited greater butanol tolerance than WT. Meanwhile, it was observed that the cytoplasm of B8 shrank in the presence of butanol. Occasionally, invaginated bodies (Fig. 3d) appeared in B8 and the inner membrane of B8 cells was not broken or leaky under butanol treatment. The huddling cytoplasm in the inner membrane could be a self-protection mechanism which protects the cells from damage due to the solvent [29]. Aono and coworkers also reported a similar phenomenon in *E. coli* cell membrane or cytoplasm in the presence of n-hexane or cyclohexane [30]. Overall, butanol-tolerant mutant B8 demonstrated a stronger stress response capability. Our results suggest that microbial cells could adapt to solvent stress via morphology change.

### DNA microarrays and data analysis

Organic solvent tolerance-related genes are usually correlated in a precise regulatory network. The global gene expression profile of mutant B8 and WT was analyzed by DNA microarray. Data were analyzed by Gene Spring Software (Santa Clara, CA, USA) to identify differentially expressed genes. For correlation of gene expression difference under solvent challenge, an over twofold change in gene expression was required with a *p* value <0.05 [31]. The results show that 329 genes (including 197 up-regulated and 132 down-regulated) exhibited differential expression (*p* < 0.05; FC ≥ 2) between B8 and WT under 0.8 % (v/v) butanol stress (Additional file 2: Figure S2). Supporting information on the detailed description of differentially expressed genes is provided in Additional file 3: Table S3. Furthermore, genes showing significant difference in transcription level were selected for clustering analysis, which helps to understand the relationships and discrepancy of samples more comprehensively and intuitively (Additional file 4: Figure S3). The same types of genes were gathered in a cluster with similar biological functions.

To identify the function of differentially expressed genes, their biological pathways corresponding to their functional class were summarized by KEGG (Table 1). These genes mainly involve in cell envelope biogenesis, carbohydrate metabolism, energy metabolism, nucleotide metabolism and two-component signal transduction system. As shown in Table 1, most genes (such as *ybbQ, glxR, hyi, gcl, glcB, glcD, glcF*) involved in glyoxylate and dicarboxylate metabolism pathway were up-regulated for over fivefold, and other genes were also enhanced by more than twofold. The glyoxylate and dicarboxylate metabolism pathway could generate ATP, and its intermediate metabolite NADH could assist in regulating proton gradient and membrane potential. This might be a key factor for improving butanol tolerance, because energy supply is essential for overcoming butanol pressure [32]. In addition, genes related to the ABC transporter pathway were also significantly up-regulated by 2.18- to 5.63-fold. ABC transporter systems consist of different transmembrane protein components and share a common ATP-binding site. ABC transporters play roles in bacterial virulence, cell growth and development, and survival under various environments [33, 34]. Based on our microarray data, ABC transporters annotated as molybdate/arginine/ferric/phosphate type transporters were identified (Table 1), which are responsible for transporting molybdate/arginine/ferric/phosphate substrates across biological membranes. Ferric is important for the regulation of intracellular redox respiratory system, while molybdate/arginine/phosphate substrates are essential for the cellular anabolism and pH homeostasis [35–38]. Therefore, these ABC transporters could affect the adaptation of *E. coli* strains under severe environments, such as solvent challenge.

**Table 1:** KEGG biological pathways of differentially expressed genes

KEGG pathway	Gene	Expression difference	Description
ABC transporters	*modA*	2.28↓	Molybdate-binding periplasmic protein precursor
	*alsB*	2.83↑	Putative LACI-type transcriptional regulator
	*artM*	2.18↓	Arginine 3rd transport system permease protein
	*fepC*	2.05↑	ATP-binding component of ferric enterobactin transport
	*fepD*	5.63↑	Ferric enterobactin transport system permease protein
	*pstS*	2.57↑	Periplasmic phosphate-binding protein
Ascorbate and aldarate metabolism	*sgaE*	2.08↑	Putative epimerase
	*ulaF*	2.56↑	Putative epimerase
	*ulaA*	2.65↑	Hypothetical protein
	*ECs5173*	2.64↑	Putative hexulose-6-phosphate isomerase
Glyoxylate and dicarboxylate metabolism	*aceA*	2.97↑	Isocitrate lyase
	*ybbQ*	7.51↑	2-Hydroxy-3-oxopropionate reductase
	*glxR*	9.86↑	Putative oxidoreductase
	*aceB*	3.02↑	Malate synthase A
	*acnA*	3.04↓	Aconitate hydrase 1
	*hyi*	9.53↑	Glyoxylate-induced protein
	*gcl*	10.60↑	Glyoxylate carboligase
	*glcB*	7.86↑	Malate synthase G
	*glcD*	12.78↑	Glycolate oxidase subunit D
	*glcF*	22.60↑	Glycolate oxidase iron–sulfur subunit
Oxidative phosphorylation	*atpC*	2.00↑	Membrane-bound ATP synthase
	*cydA*	2.07↑	Cytochrome d terminal oxidase
	*cydB*	2.18↑	Cytochrome d terminal oxidase polypeptide subunit II
Pentose phosphate pathway	*tktA*	2.31↑	Transketolase 1
	*gntK*	2.53↑	Gluconokinase 2
	*talA*	2.36↓	Transaldolase A
	*prs*	2.61↑	Phosphoribosylpyrophosphate synthetase
	*ECs3810*	2.19↑	Transketolase 1 isozyme
Phenylalanine metabolism	*mhpD*	2.24↑	2-Keto-4-pentenoate hydratase
	*mhpC*	2.41↑	2-Hydroxy-6-ketonona-2,4-dienedioic acid hydrolase
Propanoate metabolism	*tdcD*	6.46↑	Putative kinase
	*tdcE*	6.81↑	Probable formate acetyltransferase 3
	*prpD*	3.39↓	Orf, hypothetical protein
	*prpB*	3.72↓	Putative phosphonomutase 2
	*accC*	2.48↑	Acetyl CoA carboxylase
	*accB*	2.23↑	BCCP subunit; carrier of biotin
	*ECs3995*	6.59↑	Putative kinase
Pyruvate metabolism	*poxB*	3.06↓	Pyruvate oxidase
	*glcB*	7.86↑	Malate synthase G
	*accC*	2.48↑	Acetyl CoA carboxylase
	*accB*	2.23↑	BCCP subunit; carrier of biotin
	*gloB*	2.08↓	Probable hydroxyacylglutathione hydrolase
	*aceB*	3.02↑	Malate synthase A
	*pps*	2.12↑	Phosphoenolpyruvate synthase
	*tdcE*	6.81↑	Probable formate acetyltransferase 3
	*ECs0957*	3.03↓	Pyruvate oxidase
Ribosome	*rpsQ*	2.22↑	30S ribosomal subunit protein S17
	*rplQ*	2.01↑	50S ribosomal subunit protein L17
	*rpmA*	2.01↑	50S ribosomal subunit protein L27
	*rplU*	2.28↑	50S ribosomal subunit protein L21
	*rpmD*	2.04↑	50S ribosomal subunit protein L30
Starch and sucrose metabolism	*ybhC*	2.06↑	Putative pectinesterase
	*treF*	2.36↓	Cytoplasmic trehalase
	*malP*	2.19↓	Maltodextrin phosphorylase
	*otsA*	2.32↓	Trehalose-6-phosphate synthase
Two-component system	*rcsF*	2.40↑	Protein rcsF
	*ompC*	2.07↑	Outer membrane protein 1b
	*pstS*	2.57↑	High-affinity phosphate-specific transport system
Ubiquinone and other terpenoid-quinone biosynthesis	*entC*	3.10↑	Isochorismate hydroxymutase 2
	*menE*	7.17↓	o-Succinylbenzoate-CoA ligase
	*ubiX*	2.24↑	3-Octaprenyl-4-hydroxybenzoate carboxy-lyase
	*menC*	9.10↓	o-Succinylbenzoyl-CoA synthase
Phenylalanine. tyrosine and tryptophan biosynthesis	*trpE*	3.15↑	Anthranilate synthase component I
	*trpD*	5.04↑	Anthranilate synthase component II
	*aroH*	2.02↑	3-Deoxy-D-arabinoheptulosonate-7-phosphate synthase
	*ECs1836*	2.97↑	Anthranilate synthase component I
	*ECs1833*	2.46↑	Tryptophan synthase beta protein

“↑” Represents up-regulated, “↓” represents down-regulated

### Identification of genes associated with butanol tolerance

#### Analysis of membrane-related down-regulated genes

**Fig. 4:**
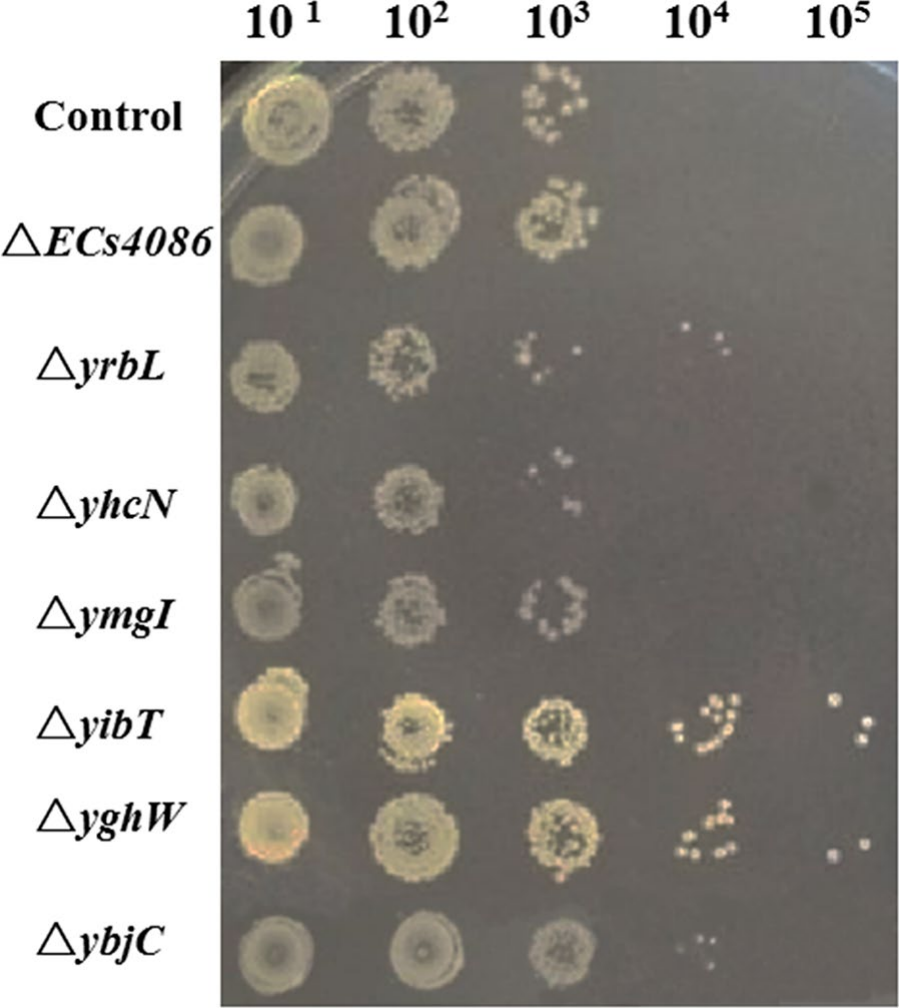
Colony-forming efficiency of *E. coli* knockout strains on LB agar. Seven single-gene knockouts were cultured in LB medium, and 0.8 % n-butanol was added at OD_660_ 0.8 followed by incubation for 1.5 h. Then, cell cultures were serially diluted at tenfold gradient, and 10 μL of the diluted solution was spotted onto LB agar for incubation at 37 °C overnight. *E. coli* JM109 was used as the control

Many genes have been confirmed to be related to the organic solvent tolerance of the *E. coli* strain. For example, overexpression of *marA* could enhance the function of AcrAB-TolC efflux pump, so that the toxic substances could be extruded more efficiently [21, 39]. *Escherichia coli* mutant △*lon* (cell envelop related gene) showed enhanced solvent tolerance level [40]. An *E. coli* mutant with gene disruptions in both *proV* and *marR* showed increased solvent tolerance due to their functions in regulation of osmotic pressure [22]. In this study, seven down-regulated genes (*yibT, yghW, ymgI, yhcN, yrbL, ECs4086, ybjC*) were selected for further study. They were rarely investigated previously and exhibited over sixfold changes in microarray analysis. All seven genes were annotated as predicted protein or hypothetical protein. The down-regulation folds of these genes are 46.72, 13.00, 16.02, 14.74, 14.07, 12.91 and 8.73, respectively. The knockout strains were constructed for further investigation. Among them, △*yibT,* △*yghW* and △*ybjC* exhibited higher n-butanol tolerance, while other knockouts showed similar growth to the control (Fig. 4). So far, there has been no report on the solvent tolerance-related functions of *yghW* and *yibT*. It was, however, noticed that these two predicted proteins are closely related to membrane proteins in stitch networks (http://www.stitch.embl.de/). Stitch is a database which contains interaction information for connected proteins and chemicals. It allows querying by genes name and metabolic pathways [41]. In stitch networks, *gnsA* and *gnsB* are predicted regulators of phosphatidylethanolamine synthesis and unsaturated fatty acids regulatory proteins, respectively, which are both linked to *yibT*. Gene *yghW* is linked to the inner membrane protein encoding genes *ybjO* and *ybjM*, as well as genes encoding the predicted lipoprotein or conserved protein in stitch networks. It is therefore presumed that modulation of membrane fatty acid compositions is one possible defense mechanism of *yibT* and *yghW*. It has been reported that the properties of membrane fatty acids, such as fatty acid chain length, branching pattern and unsaturation degree of fatty acids, could change when exposed to environment stress [42–45]. In this study, phospholipids of △*yghW* and △*yibT* were extracted and analyzed. Our results show that the main components of membrane fatty acids are C16:1, C16:0, C18:0 and C18:1, which account for over 70 % of the total fatty acids in all strains (Table 2) and are responsible for the integrity and fluidity of the membrane [46]. It was noticed that the proportion of unsaturated fatty acid (UFA) in total fatty acids was increased in both knockouts, especially palmitoleic acid (C16:1) and oleic acid (C18:1). Oleic acid (C18:1) has been regarded as the most important UFA in counteracting the toxic effects of the solvent by modulating plasma membrane fluidity [47]. Additionally, palmitoleic acid (C16:1) could influence the rigidity and integrity of membrane lipid bilayer [46, 48]. It is speculated that a higher proportion of oleic acid and palmitoleic acid in △*yghW* and △*yibT* contributes to a lower membrane fluidity, higher rigidity and membrane integrity, which might be a compensatory advantage of the membrane challenged by butanol. As a result of changes in membrane fatty acid composition, other physicochemical properties of the membrane, such as proton permeability and lipid–protein interactions could also be affected [49, 50]. Our findings suggest that the butanol tolerance mechanism of △*yghW* and △*yibT* are related to membrane fatty acid composition.

**Table 2:** Comparison of fatty acid composition of knockouts and control

Major fatty acids	Fatty acid (%)^a^			Difference △*yibT*	*p* value^b^	Difference △*yghW*	*p* value^b^
	JM109	△*yibT*	△*yghW*				
C11:0	0.20 ± 0.04	0.00 ± 0.00	0.13 ± 0.04	−0.20	***	−0.08	**
C12:0	0.69 ± 0.44	0.33 ± 0.04	0.45 ± 0.12	−0.36	*	−0.25	*
C13:0	0.00 ± 0.00	0.05 ± 0.07	0.07 ± 0.09	0.05	*	0.07	*
C14:0	2.24 ± 0.96	1.42 ± 0.27	1.41 ± 0.13	−0.82	*	−0.84	*
C15:0	0.65 ± 0.52	2.45 ± 0.05	3.03 ± 0.87	1.80	***	2.38	***
C15:1	0.00 ± 0.00	0.12 ± 0.16	0.31 ± 0.07	0.12	*	0.31	***
C16:0	26.96 ± 1.32	18.86 ± 0.33	15.48 ± 1.18	−8.10	***	−11.49	***
C16:1	9.06 ± 1.08	10.90 ± 1.05	11.08 ± 1.52	1.84	**	2.02	*
C17:0	1.52 ± 0.42	6.18 ± 0.77	5.67 ± 0.37	4.66	***	4.15	***
C17:1	4.69 ± 0.62	4.80 ± 0.39	6.35 ± 0.88	0.11	*	1.66	**
C18:0	9.56 ± 0.95	6.49 ± 1.44	5.36 ± 0.31	−3.07	**	−4.20	***
C18:1	21.03 ± 1.36	25.23 ± 0.73	27.05 ± 0.37	4.20	***	6.02	***
C18:2	0.03 ± 0.04	0.06 ± 0.08	0.42 ± 0.29	0.03	*	0.39	**
C18:3	0.38 ± 0.54	0.00 ± 0.00	0.04 ± 0.06	−0.38	*	−0.34	*
C19:1	1.22 ± 0.17	0.08 ± 0.11	0.07 ± 0.09	−1.14	***	−1.16	***
C20:0	0.23 ± 0.01	0.08 ± 0.11	0.16 ± 0.06	−0.15	**	−0.08	*
C20:1	1.07 ± 0.62	0.00 ± 0.00	0.00 ± 0.00	−1.07	**	−1.07	**
C22:1	0.13 ± 0.18	0.06 ± 0.08	0.05 ± 0.07	−0.07	*	−0.08	*
C25:0	0.06 ± 0.08	0.07 ± 0.09	0.00 ± 0.00	0.01	*	−0.06	*
SFA	42.09 ± 0.68	35.91 ± 0.08	31.73 ± 1.92	−6.18	***	−10.37	***
UFA	37.59 ± 0.51	41.22 ± 2.28	45.36 ± 3.34	3.63	**	7.76	***

^a^The values represent percentages of total fatty acids and are means ± standard deviations from three independent experiments

^b^Statistical significance between knockout and control strain (*E.coli JM109*)

* *p* > 0.05, ** 0.01 < *p* < 0.05, *** *p* < 0.01

Moreover, the hydrophobicity, acidity and alkalinity of cell surface are important properties related to the solvent tolerance [51–53]. Using the MATS method, the adsorptions of three strains (△*yghW*, △*yibT* and JM109) to organic solvents with different hydrophobicities such as chloroform, hexadecane, ethyl acetate and decane were determined (Additional file 5: Figure S4). Our results show that the adsorptions of △*yghW*, △*yibT* and JM109 to chloroform were 1.89-, 1.86- and 1.61-fold of those to hexadecane, indicating that the alkaline strength of the knockout strains was higher than that of WT. The alkaline strength of the cell surface has been reported to be proportional to the adsorption ratio of chloroform adsorption to hexadecane [51]. Similarly, the adsorptions of △*yghW*, △*yibT* and JM109 to ethyl acetate were 1.81-, 1.79- and 1.65-fold of those to decane. It has also been reported that the acidity strength of cell surface was proportional to the adsorption ratio of ethyl acetate to decane [51]. It is supposed that the increase of surface acidity and alkalinity is due to the presence of proteins and charged chemical groups on the cell surface, such as PO_4_^2−^ and COO^−^ [54], which may assist strains to counteract extracellular toxic substances. Additionally, the adhesion to hexadecane and decane reflects the hydrophobicity of the cell surface. In Additional file 5: Figure S4, the adhesion of △*yghW* and △*yibT* cells to hexadecane and decane was weaker compared with that of the control, suggesting that the surface hydrophobicity of the knockouts was higher than that of the control. The contact angle measurement (CAM) was also conducted to determine the surface hydrophobicity. Our results show that the contact angle of the control, △*yghW* and △*yibT* were 21.17 ± 1.78°, 36.25 ± 2.13° and 34.65 ± 2.04°, respectively (Additional file 6: Figure S5). Consistent with the results of MATS, CAM assay represents a higher surface hydrophobicity of △*yghW* and △*yibT*. Other studies suggest that the expansion of surface hydrophobic region could promote the interactions between the phospholipids and embedded proteins and bonds between cation and electronegative phospholipids in the membrane [55, 56]. It is therefore rational to conjecture that the cell surface of △*yghW* and △*yibT* is less permeable, which could help to prevent the intruding of toxicity compounds.

**Fig. 5:**
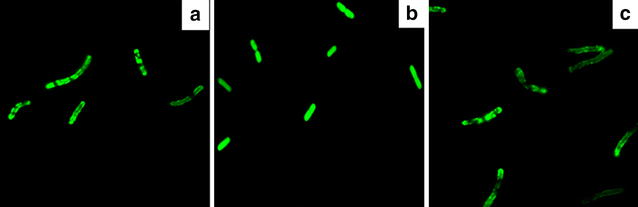
Fluorescent micrographs of recombinant *E. coli* cells expressing **a** YbjC–PPP–GFP fusion protein, **b** GFP protein (negative control) and **c** YidC–PPP–GFP fusion protein (positive control) (1000× magnification)

The gene (*ybjC*) encoding a predicted inner membrane protein was also investigated. To verify the location of YbjC and its tolerance-related mechanism, YbjC–GFP (green fluorescent protein) fusion protein was constructed. As shown in Fig. 5a, a clear fluorescence signal surrounding the membrane region was detected in *E. coli* cells expressing fusion protein YbjC–GFP. Differently, the entire cells were filled with green fluorescence when GFP was expressed alone (Fig. 5b). Here, a known membrane protein YidC fused with GFP was expressed as the positive control (Fig. 5c), and the microscopy pattern of YbjC–GFP fusion was similar to YidC–GFP. This further suggests that YbjC is a membrane protein. Unfortunately, the function of YidC related to the butanol tolerance mechanism is not clear yet.

#### Analysis of carboxylic acid metabolism-related up-regulated genes

Gene cluster *glc*, encoding enzymes involved in glyoxylate and dicarboxylate metabolism, was up-regulated significantly in this study. For example, genes *glcF, glcA, glcD,glcG, glcB* and *gcl* were up-regulated by 22.60-, 18.17-, 12.78-, 12.75- 7.86- and 10.60-fold, respectively. The above genes and the *glc* gene cluster were selected for overexpression to investigate their functional relevance to butanol tolerance. As shown in Additional file 7: Figure S6, *glcA* overexpression strain could barely grow even without butanol. For *glcB*, *glcD* and *glcG* overexpression strains, similar growth with control strain (JM109 carrying empty plasmid pQE80L) was observed in the presence and absence of butanol. Strains overexpressing *glcF* and *gcl* showed improved cell growth compared with the control. Furthermore, *glcDEFGBA* cluster co-expression strains also exhibited better growth than the control with or without butanol. The possible explanation is that *glcA* encodes glycolate transporter, and its overexpression could result in metabolism imbalance of carbon source and energy in cells [57]. *glcB* encodes malate synthase G which is not essential for growth. *glcD* and *glcG* encode different subunits of glycolate oxidase, which have no significant effect on the oxidase activity [58]. Gene *glcF* encodes iron–sulfur subunit of glycolate oxidase that catalyzes the conversion of glycolate to glyoxylate, and *gcl* encodes glyoxylate carboligase that transforms glyoxylate into phosphoglycerate [59, 60]. One possible explanation is that these two enzymes, glycolate oxidase and glyoxylate carboligase, directly improve the TCA cycle via modulating glyoxylate and pyruvate, which replenish metabolic intermediates for the TCA cycle (Additional file 8: Figure S7). In this study, *rpoD* mutagenesis approach conduced to enhanced OST phenotype by regulating the expression level of hundreds of genes. Our results also suggest that the co-expression of *glc* gene cluster is more favorable for cell growth than individual gene overexpression, which might have minor or negative effect on butanol tolerance. To elucidate the possible mechanism, the concentrations of carboxylic acids in TCA cycle were monitored in *glcF* and *gcl* overexpression strains. For both strains, pyruvate concentrations were increased in the presence of butanol, whereas higher pyruvate levels were also observed without butanol (Additional file 9: Table S4). Moreover, the fumarate concentration was also increased in the overexpression strains without butanol, but declined slightly when 0.8 % (v/v) butanol was added. This phenomenon may be attributed to the inhibition of certain TCA cycle enzymes (such as succinate dehydrogenase) that are sensitive to the oxidative stress induced by butanol. A similar phenomenon was reported by Fu and coworkers [32]. Although the elevated pyruvate level in *gcl and glcF* overexpression strains does not seem to be directly related to butanol stress, it is assumed that the up-regulation of these two genes is favorable for the cell growth by replenishing TCA metabolic intermediates.

## Conclusions

In summary, we successfully isolated an *E. coli* strain harboring *rpoD* mutant B8 with 2 % (v/v) butanol tolerance using global transcriptional machinery engineering approach. Based on DNA microarrays results, 329 genes (including 197 up-regulated and 132 down-regulated) showed over twofold difference in the expression level compared with WT after butanol treatment. These genes are mainly involved in the metabolic pathways including ABC pump, ascorbate and aldarate metabolism, energy metabolism, two-component signal transduction system and amino acid metabolism. Tolerance mechanisms of several critical genes have been elucidated. Among them, down-regulated genes *yghW* and *yibT* were proved to improve n-butanol tolerance due to their regulatory roles in membrane fatty acids composition. YbjC was confirmed to be a membrane protein, while up-regulated genes *gcl* and *glcF* could replenish TCA cycle metabolic intermediates to improve cell growth and metabolism. These results could provide the potential approach for the construction of *E. coli* strain as a bio-butanol producer.

## Methods

### Strains, plasmids and culture conditions

*Escherichia coli* JM109 was used as the host strain. Gene deletion strains were generated by Red-mediated recombination approach and overexpression strains were generated using pQE80L as an expression vector. Strains and plasmids are listed in Additional file 10: Table S1. Primers used in this study are listed in Additional file 11: Table S2. Plasmid pQE80L was purchased from Qiagen GmbH (Hilden, Germany). pHACM-*rpoD*^WT^ was presented as a kind gift of Dr. Huimin Yu (Tsinghua University, China). Restriction enzymes and PrimeSTAR^®^HS DNA Polymerase were purchased from Takara (Tokyo, Japan). The dam-methylated DNA-specific restriction enzyme *Dpn*I was purchased from New England Biolabs (Ipswich, MA, USA). GeneMorph II Random Mutagenesis Kit was obtained from Stratagene (La Jolla, CA, USA).

All strains were grown in Luria–Bertani (LB) medium (tryptone 10 g/L, yeast extract 5 g/L, NaCl 10 g/L) at 37 °C, 120 rpm. Butanol was added as specified in each experiment. When necessary, antibiotics chloramphenicol (34 μg/mL), ampicillin (100 μg/mL) and kanamycin (50 μg/mL) were added to the media. For gene overexpression, 0.2 mM IPTG was added to the medium at around 0.3 OD_660_.

### Construction of random mutagenesis library

Using plasmid pHACM-*rpoD*^WT^ as the template, random mutagenesis was performed by the GenemorphII Random Mutagenesis kit (Stratagene) with a mutation rate of approximately 4.5–9 mutations/kb. The error-prone PCR program was set as follows: 5 min at 95 °C, 30 cycles of 95 °C for 30 s, 57 °C for 1 min, followed by 72 °C for 2 min, and 10 min at 72 °C. After running the whole plasmid PCR, the PCR product mixture was digested with *Dpn*I and then transformed into *E. coli* JM109. *Escherichia coli* transformants were plated on LB agar plates containing 34 μg/mL chloramphenicol and incubated at 37 °C. Then the colonies were scraped off to create a σ^70^ mutant library for further butanol-tolerant phenotype selection. The total mutant library size was approximately 10^6^.

### Phenotype selection of n-butanol-tolerant mutants

#### The first round of screening

First, 483 colonies were selected from the σ^70^ mutant library on agar plate containing 0.5 % butanol (v/v). Then, these colonies were inoculated into 24-well plates with LB/Cm liquid medium. Butanol (0.5 %, v/v) was added into the culture when OD_660_ reached 0.2, and the cells were further incubated for 8 h at 120 rpm and 37 °C. Cell density was measured, and ten mutants with OD_660_ above 1.0 were selected. Then, the selected mutants were cultured in a medium containing higher concentrations (0.5–1.5 %, v/v) of n-butanol by 0.1 % (v/v) gradient, until a strain exhibiting the highest tolerance was obtained. The plasmid harboring the *rpoD* mutant was then sequenced and designated as D3.

#### The second round of screening

To further improve the n-butanol tolerance, σ^70^ mutant D3 was used as a template to construct a random mutagenesis library for a second round of screening. The screening method was the same as that in the first round. The best mutant B8 was selected and evaluated under higher n-butanol concentrations (0.1 % gradient increasing from 1.2 to 2.2 %) to determine its maximum tolerant level.

### Solvent shock treatment

The overnight cell culture was inoculated (1 %) into a fresh LB medium. When OD_660_ reached 0.8, 3 % (v/v) n-butanol was added into the culture. After incubation at 37 °C for 1.5 h, the cultures were serially diluted for 10^5^, 10^4^, 10^3^, 10^2^ and 10-fold with aseptic water. Then, 10 μL of the each diluted culture was spotted onto LB/agar plates and further incubated at 37 °C overnight.

### Cell morphology

The σ^70^ mutant B8 and WT strains were cultured overnight and inoculated (1 %) into fresh LB/Cm liquid medium for incubation at 37 °C and 120 rpm for 8 h with or without 0.8 % (v/v) n-butanol. The cells were diluted and spread on LB/Cm agar plates. Single colonies were treated as described in the literature and observed using transmission electron microscope [30]. Briefly, single colonies were picked and fixed by immersion in 2.5 % glutaraldehyde at 4 °C for 3 h. The cell suspension was mixed once every half-hour. Then, cells were washed four times with 0.1 M phosphate buffer (pH 7.2), and the samples were diluted for cell morphology observation under Hitachi-H7650 transmission electron microscopy (Japan). The average cellular size of WT and B8 was counted on the electron microscope based on around 100 cells.

### DNA microarrays

*Escherichia coli* strains harboring σ^70^ mutant B8 and WT were cultured overnight and inoculated (1 %) into fresh medium. n-Butanol (0.8 %, v/v) was added at 0.8 OD_660_ and further incubated for 1.5 h. Cells were harvested by centrifugation (8800 *g*, 4 °C). Total RNA was extracted using Qiagen RNeasy kit (Hilden, Germany) following the manufacturer’s instructions. Qualified total RNA was further purified by Qiagen RNeasy mini kit and RNase-Free DNase Set. Three biological replicates of RNA samples were stored in dry ice and subjected to further DNA microarray analysis. The microarray service was provided by Shanghai Biotechnology Co., Ltd. (Shanghai, China) using Agilent SurePrint *E. coli* 8 × 15 K slides, and the quality and integrity of RNA were examined before analysis.

Slides were scanned by Agilent Microarray Scanner (Santa Clara, CA, USA), and data were extracted with Agilent Feature Extraction software 10.7. Raw data were normalized by Quantile algorithm, Gene Spring Software 11.0 (Santa Clara, CA, USA). Differentially expressed genes were identified using the rank product method [61]. The MeV (TM4) software was used for clustering and other expression profile analysis [62]. The related metabolic pathway of differentially expressed genes was analyzed using Kyoto Encyclopedia of Gene and Genomics (KEGG) database [63]. The microarray data have been deposited at the gene expression omnibus (GEO) under the accession number GSE79305.

### Assay of n-butanol sensitivity of knockout and overexpression strains

Gene knockout (for down-regulated genes) and overexpression (for up-regulated genes) strains were constructed based on the microarrays results. Spot assay as a tolerance confirmation method was conducted for knockout strains. n-Butanol (0.8 %, v/v) was added when the cell density reached 0.8 OD_660_, then the cells were further cultured for 1.5 h. Cell culture was serially diluted at a tenfold gradient, and 10 μL of the diluted culture was spotted onto LB/agar plates for incubation at 37 °C overnight. For overexpression strains, the overnight culture was inoculated (1 %) into fresh medium and cultured at 37 °C and 120 rpm, and 0.2 mM IPTG was added when OD_660_ reached 0.3. Then, 0.8 % (v/v) butanol was added at 0.8 OD_660_ for further incubation at 30 °C for 8 h. The growth of the strains was monitored by measuring cell density at OD_660_.

### Lipid extraction

Lipid extraction was performed as described by Bligh [64]. Briefly, the stationary phase cells of *E. coli* JM109, △*yibT* and △*yghW* were collected by centrifugation and washed with 10 mM, pH 7.4 sodium phosphate buffer. The cell pellets (0.3 g wet weight) were suspended in a mixture consisting of buffer, methanol and chloroform in the proportion of 0.8:2:1 (v/v) and incubated at room temperature for 2 h, with brief shaking every half-hour. Then, the extract was centrifuged at 4 °C after diluting with methanol and chloroform (1:1, v/v). The lower chloroform phase (containing lipids) was added into the methanol solution containing 5 % (v/v) H_2_SO_4_. After 2 h of methylation at 70 °C, the mixture was cooled to room temperature and extracted three times with pentane. Then, the samples were air dried to remove pentane. Finally, the samples were reconstituted with n-hexane for GC–MS analysis.

### Determination of cell surface hydrophobicity

MATS (microbial adhesion to solvents) analysis was performed following the MATH method developed by Bellon-Fontaine with slight modification [53, 65]. Briefly, the strains were cultured in LB medium, and butanol (0.8 %, v/v) was added when OD_660_ reached 0.8. Cells were further incubated for 12 h at 37 °C and were collected by centrifugation (8800* g* and 10 min at 4 °C), then washed with 100 mM, pH 6.0 potassium phosphate buffer and centrifuged again. The cell concentration was then adjusted to around 1.0 OD_400_ (A_0_) using the same potassium phosphate buffer. The cell suspension (4.8 mL) was mixed with 0.8 mL of chloroform, hexadecane, ethyl acetate and decane, respectively. The two-phase mixture was mixed by vortexing for 90 s and then incubated statically at room temperature for 15 min. The aqueous phase was removed and OD_400_ was measured (A) to calculate the adhesion ratio [Adhesion % = (1 − A/A_0_) × 100 %]. The above experiment was repeated for three times.

Contact angle measurements (CAM) was performed to measure the surface hydrophobicity [51]. Briefly, strains were cultured in the LB medium, and butanol (0.8 %, v/v) was added when OD_660_ reached 0.8. Cells were further incubated for 12 h at 37 °C and were collected by centrifugation at 8800 *g* for 10 min at 4 °C and washed twice by 0.85 % saline. Then, the concentration of cell suspension was adjusted to 50 mg wet cells/mL, and 10 mL cells suspension was filtered through polyvinylidene difluoride membrane (0.22 μm, 50 mm in diameter) under vacuum. For each strain, three biological replicates were performed and measured independently. The contact angle was measured in three phases: the bacterial lawn, n-tetradecane and a droplet of distilled water using a contact angle meter (Dataphysics, Germany).

### Analytical methods

#### Fatty acid component analysis

The fatty acid component analysis was performed as previously described [66, 67]. The fatty acid profile was assessed with Trace1310 GC equipped with TSQ8000 MSD (Thermo Fisher Scientific, Massachusetts, USA) and an HP-5MS methylpolysiloxane phase column (30 m × 0.25 mm × 0.25 μm). The GC conditions are as follows: the initial temperature of 60 °C for 2 min, 8 °C/min to 150 °C, then to 250 °C at a rate of 3 °C/min, finally at 10 °C/min to 280 °C and held for 5 min. Fatty acids and other volatile compounds were identified by mass spectral library search.

#### Quantification of pyruvate and fumarate

Overexpression strains and control (JM109 carrying empty plasmid pQE80L) were cultured overnight and inoculated into fresh LBG medium (1 %, v/v). IPTG (0.2 mM) was added at 0.3 OD_660_, cells were further cultured for 8 h with or without 0.8 % n-butanol. The cell culture was centrifuged and the supernatant was subjected to HPLC analysis using Agilent 1260 (Palo Alto, USA) equipped with an Agilent Hi-Plex H PL1170-6830 column (7.7 × 300 mm, 8 µm) at 55 °C. 5 mM H_2_SO_4_ was used as an eluent at a flow rate of 0.6 mL/min.

## Additional file

10.1186/s13068-016-0527-9 Cell growth of σ^70^ mutants and WT in the presence of 1.2 % (v/v) n-butanol. All mutant strains were cultured in 24-well plates at 37 °C for 8 h, 1.2 % (v/v) n-butanol were added at 0.2 OD_660_.
10.1186/s13068-016-0527-9 Volcano plot of global gene expression differences between σ^70^ mutant B8 and WT upon butanol challenge. X axis: Log_2_ (Fold Change); Y axis: -Log10 (p-value). Green lines parallel to X axis represents p-value = 0.05. The left red zone denotes the number of down-regulated genes; the right red zone denotes the number of up-regulated genes (p < 0.05; FC ≥ 2).
10.1186/s13068-016-0527-9 Genes significantly changed (fold change ≥ 2, p-value < 0.05) in B8 vs control with 0.8 % (v/v) butanol treatment.
10.1186/s13068-016-0527-9 Transcription profile analysis of genes exhibiting significantly different expression levels in σ^70^ mutant B8 and WT. Hierarchical clustering using differentially expressed genes (probe sets) (p < 0.05; FC ≥ 2). Abscissa represents samples of B8 and WT, the ordinate represents different genes. Three biological replicates were performed.
10.1186/s13068-016-0527-9 Analysis of surface hydrophobicity of *E. coli* knockout strains △*yghW*, △*yibT* and JM109 (control) by MATS. Three biological replicates were performed.
10.1186/s13068-016-0527-9 Contact angle measurement of *E. coli* knockout strains (A) JM109 (control), (B) △yghW, and (C) △yibT. Three biological replicates were performed.
10.1186/s13068-016-0527-9 Analysis of n-butanol tolerance of various gene overexpression strains with or without 0.8 % n-butanol. Strains were pre-cultured at 37 °C with addition of 0.2 mM IPTG at 0.3 OD_660_. Then 0.8 % (v/v) butanol was added at 0.8 OD_660_ for further incubation at 30 °C for 8 h. Three biological replicates were performed.
10.1186/s13068-016-0527-9 Pathways of glycolate metabolism and tricarboxylic acid cycle in Escherichia coli.
10.1186/s13068-016-0527-9 Pyruvate and fumarate levels in cell cultures of gcl and glcF overexpression strains with or without butanol.
10.1186/s13068-016-0527-9 Strains and plasmids used in this study.
10.1186/s13068-016-0527-9 Primes used in this study.

## Abbreviations

gTME: global transcription machinery engineering
KEGG: Kyoto Encyclopedia of Gene and Genomics
UFA: unsaturated fatty acid
SFA: saturated fatty acid
MATS: microbial adhesion to solvents
CAM: contact angle measurement
GFP: green fluorescent protein
