# Supplementary material for: DNA microarray of global transcription factor mutant reveals membrane-related proteins involved in n-butanol tolerance in Escherichia coli
Source: Biotechnol Biofuels. 2016 Jun 1;9:114. doi: 10.1186/s13068-016-0527-9 (PMC4888631; doi:10.1186/s13068-016-0527-9)
Supplement: Supplementary file 1 — 10.1186/s13068-016-0527-9 Cell growth of σ70 mutants and WT in the presence of 1.2 % (v/v) n-butanol. All mutant strains were cultured in 24-well plates at 37 °C for 8 h, 1.2 % (v/v) n-butanol were added at 0.2 OD660. [file 13068_2016_527_MOESM1_ESM.docx]

**DNA Microarray of Global Transcription Factor Mutant Reveals Membrane-Related Proteins Involved in n-Butanol Tolerance in *Escherichia coli***

# Supplementary Online Material

**Additional file 1**. Cell growth of σ^70^ mutants and WT in the presence of 1.2% (v/v) n-butanol. All mutant strains were cultured in 24-well plates at 37°C for 8 h, 1.2% (v/v) n-butanol were added at 0.2 OD_660_. (Fig. S1)

**Fig. S1**
