# Supplementary material for: DNA microarray of global transcription factor mutant reveals membrane-related proteins involved in n-butanol tolerance in Escherichia coli
Source: Biotechnol Biofuels. 2016 Jun 1;9:114. doi: 10.1186/s13068-016-0527-9 (PMC4888631; doi:10.1186/s13068-016-0527-9)
Supplement: Supplementary file 2 — 10.1186/s13068-016-0527-9 Volcano plot of global gene expression differences between σ70 mutant B8 and WT upon butanol challenge. X axis: Log2 (Fold Change); Y axis: -Log10 (p-value). Green lines parallel to X axis represents p-value = 0.05. The left red zone denotes the number of down-regulated genes; the right red zone denotes the number of up-regulated genes (p < 0.05; FC ≥ 2). [file 13068_2016_527_MOESM2_ESM.docx]

**DNA Microarray of Global Transcription Factor Mutant Reveals Membrane-Related Proteins Involved in n-Butanol Tolerance in *Escherichia coli***

# Supplementary Online Material

**Additional file 2**. Volcano plot of global gene expression differences between σ^70^ mutant B8 and WT upon butanol challenge. X axis: Log_2_ (Fold Change); Y axis: -Log10 (*p*-value). Green lines parallel to X axis represents *p*-value=0.05. The left red zone denotes the number of down-regulated genes; the right red zone denotes the number of up-regulated genes (*p*<0.05; FC≥2). (Fig. S2)


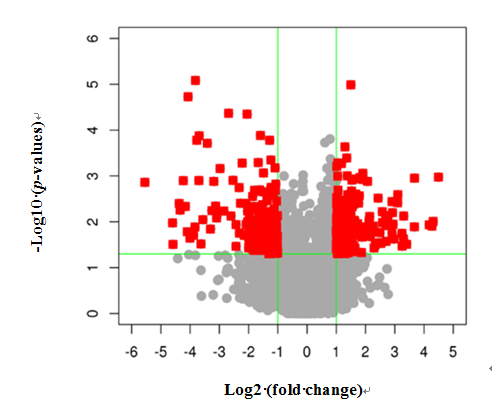


**Fig. S2**
