# Supplementary material for: DNA microarray of global transcription factor mutant reveals membrane-related proteins involved in n-butanol tolerance in Escherichia coli
Source: Biotechnol Biofuels. 2016 Jun 1;9:114. doi: 10.1186/s13068-016-0527-9 (PMC4888631; doi:10.1186/s13068-016-0527-9)
Supplement: Supplementary file 3 — 10.1186/s13068-016-0527-9 Genes significantly changed (fold change ≥ 2, p-value < 0.05) in B8 vs control with 0.8 % (v/v) butanol treatment. [file 13068_2016_527_MOESM3_ESM.docx]

**DNA Microarray of Global Transcription Factor Mutant Reveals Membrane-Related Proteins Involved in n-Butanol Tolerance in *Escherichia coli***

# Supplementary Online Material

**Additional file 3.** Genes significantly changed (fold change≥2, *p-*value<0.05) in B8 vs control with 0.8% (v/v) butanol treatment. (Table S3).

**Table S3** Genes significantly changed (fold change≥2, *p-*value<0.05) in B8 vs control with 0.8% (v/v) butanol treatment.

| Gene | Description | Fold change | *p*-values |
| --- | --- | --- | --- |
| *ccmE* | cytochrome c biogenesis, possible subunit of a heme lyase [b2197] | -2.328 | 0.033 |
| *pspA* | phage shock protein, inner membrane protein [b1304] | -2.747 | 0.024 |
| *ydjM* | orf, hypothetical protein [b1728] | -2.574 | 0.032 |
| *atpC* | membrane-bound ATP synthase, F1 sector, epsilon-subunit [b3731] | 2.005 | 0.037 |
| *ompT* | outer membrane protein 3b [b0565] | 2.915 | 0.031 |
| *talA* | transaldolase A [b2464] | -2.360 | 0.027 |
| *yjjM* | orf, hypothetical protein [b4357] | 2.180 | 0.018 |
| *poxB* | Pyruvate dehydrogenase [c_1004] | -3.030 | 0.021 |
| *yrbL* | orf, hypothetical protein [Z4570] | -13.649 | 0.000 |
|  | Hypothetical protein [c_0205] | 2.154 | 0.044 |
| *cld* | regulator of length of O-antigen component of lipopolysaccharide chains [b2027] | 2.030 | 0.022 |
| *tdcF* | orf, hypothetical protein [b3113] | 7.504 | 0.004 |
| *ECs0346* | putative transporter [ECs0346] | 2.646 | 0.009 |
| *ybiJ* | orf, hypothetical protein [b0802] | -3.256 | 0.002 |
| *napC* | cytochrome c-type protein [b2202] | -2.054 | 0.034 |
| *yfiL* | orf, hypothetical protein [b2602] | -2.458 | 0.009 |
| *ssb* | single strand binding protein [L7084] | -2.860 | 0.004 |
| *ivy* | orf, hypothetical protein [b0220] | 3.784 | 0.001 |
| *treF* | cytoplasmic trehalase [b3519] | -2.362 | 0.002 |
|  | Hypothetical protein [c_4656] | 2.041 | 0.012 |
| *marR* | multiple antibiotic resistance protein; repressor of mar operon [b1530] | -2.560 | 0.041 |
| *glpQ* | glycerophosphodiester phosphodiesterase, periplasmic [b2239] | 2.084 | 0.050 |
| *yjjM* | Hypothetical protein yjjM [c_5436] | 2.094 | 0.023 |
| *yjhQ* | orf, hypothetical protein [b4307] | 2.040 | 0.006 |
| *ymgD* | orf, hypothetical protein [b1171] | 5.342 | 0.017 |
| *fhuF* | Ferric iron reductase protein fhuF [c_5446] | 3.171 | 0.002 |
| *accC* | acetyl CoA carboxylase, biotin carboxylase subunit [b3256] | 2.477 | 0.020 |
| *groL* | GroEL, chaperone Hsp60, peptide-dependent ATPase, heat shock protein [b4143] | 2.106 | 0.044 |
|  | IS1 transposase InsAB', C-ter fragment (pseudogene) [b4564] | -2.018 | 0.021 |
|  | Hypothetical protein [c_2723] | 2.756 | 0.027 |
| *htpG* | chaperone Hsp90, heat shock protein C 62.5 [b0473] | 2.013 | 0.009 |
| *ytfL* | putative transport protein [b4218] | 2.414 | 0.015 |
| *ECs0572* | putative transport protein [ECs0572] | 19.330 | 0.012 |
| *sgaE* | putative epimerase/aldolase [Z5807] | 2.062 | 0.008 |
| *ybbT* | Ureidoglycolate hydrolase [c_0620] | 5.755 | 0.030 |
|  | Hypothetical protein [c_4113] | 2.479 | 0.031 |
| *frvX* | frv operon protein [b3898] | -2.533 | 0.008 |
| *rcsF* | regulator in colanic acid synthesis RcsF [ECs0198] | 2.452 | 0.003 |
| *ECs3995* | putative kinase [ECs3995] | 6.594 | 0.010 |
|  | single-strand binding protein | -3.505 | 0.005 |
| *rsmC* | putative enzyme [Z5972] | 2.634 | 0.023 |
| *ykgG* | orf Unknown function [Z0386] | 2.334 | 0.027 |
| *cof* | orf, hypothetical protein [b0446] | 2.003 | 0.031 |
| *ydcL* | orf, hypothetical protein [b1431] | -6.418 | 0.000 |
| *pspA* | Phage shock protein A [c_1774] | -2.848 | 0.012 |
| *chaC* | cation transport regulator [b1218] | -5.780 | 0.001 |
|  | putative L-serine dehydratase [b3112] | 7.576 | 0.006 |
| *lacZ* | Beta-galactosidase [c_0459] | -2.151 | 0.009 |
| *ECs0346* | putative transporter [ECs0346] | 2.488 | 0.018 |
| *yjiY* | Hypothetical protein yjiY [c_5429] | -3.993 | 0.013 |
|  | orf, hypothetical protein [b0510] | 5.397 | 0.003 |
|  | Hypothetical protein [c_3040] | 2.585 | 0.030 |
| *poxB* | pyruvate oxidase [b0871] | -3.059 | 0.012 |
| *accB* | acetylCoA carboxylase, BCCP subunit; carrier of biotin [b3255] | 2.227 | 0.006 |
| *ybhC* | putative pectinesterase [Z0943] | 2.056 | 0.015 |
| *ybaT* | putative amino acid/amine transport protein [b0486] | -2.888 | 0.021 |
|  | Conserved hypothetical protein [c_2930] | -3.466 | 0.002 |
| *glxR* | putative oxidoreductase [b0509] | 9.859 | 0.024 |
| *rutB* | putative synthetase [b1011] | -2.474 | 0.050 |
| *entC* | isochorismate hydroxymutase 2, enterochelin biosynthesis [b0593] | 3.102 | 0.010 |
| *hupB* | DNA-binding protein HU-beta, NS1 [b0440] | 2.001 | 0.033 |
| *yjgN* | orf, hypothetical protein [b4257] | -3.008 | 0.042 |
| *exbD* | uptake of enterochelin; tonB-dependent uptake of B colicins [b3005] | 2.943 | 0.013 |
| *finO* | fertility inhibition protein (conjugal transfer repressor) [L7001] | -3.685 | 0.027 |
| *yghW* | orf, hypothetical protein [b2998] | -46.716 | 0.001 |
| *tktA* | Transketolase 1 [c_3520] | 2.168 | 0.035 |
|  | Hypothetical protein [c_2891] | -16.759 | 0.000 |
| *glcA* | putative permease [b2975] | 18.175 | 0.012 |
| *gntK* | gluconokinase 2, thermoresistant [Z4805] | 2.533 | 0.040 |
| *aceA* | isocitrate lyase [b4015] | 2.971 | 0.041 |
|  | Zinc resistance-associated protein precursor [c_4959] | 2.019 | 0.004 |
|  | Hypothetical protein [c_4300] | -2.720 | 0.026 |
| *grpE* | phage lambda replication; host DNA synthesis; heat shock protein; protein repair [b2614] | 2.284 | 0.006 |
| *ECs3215* | putative adenine-specific methylase [ECs3215] | 2.196 | 0.023 |
| *rcsF* | regulator in colanic acid synthesis; interacts with RcsB [b0196] | 2.458 | 0.000 |
| *mhpD* | 2-keto-4-pentenoate hydratase [b0350] | 2.243 | 0.004 |
| *yiaD* | putative outer membrane protein [b3552] | 2.278 | 0.014 |
| *tdcD* | putative kinase [b3115] | 5.983 | 0.010 |
| *yhjC* | putative transcriptional regulator LYSR-type [b3521] | 2.060 | 0.016 |
| *cydA* | cytochrome d terminal oxidase, polypeptide subunit I [b0733] | 2.068 | 0.015 |
| *glyA* | serine hydroxymethyltransferase [b2551] | 2.872 | 0.002 |
| *rpsQ* | 30S ribosomal subunit protein S17 [b3311] | 2.217 | 0.021 |
| *trpE* | Anthranilate synthase component I [c_1730] | 3.030 | 0.009 |
| *lacY* | galactoside permease [b0343] | -2.590 | 0.006 |
| *mdtD* | putative transport protein [b2077] | -2.417 | 0.018 |
|  | Hypothetical protein [c_0552] | 2.167 | 0.013 |
| *ECs0957* | pyruvate oxidase [ECs0957] | -3.026 | 0.010 |
| *fhuF* | orf, hypothetical protein [b4367] | 3.665 | 0.021 |
| *rimK* | ribosomal protein S6 modification protein [b0852] | -4.839 | 0.004 |
| *malP* | maltodextrin phosphorylase [b3417] | -2.187 | 0.030 |
| *yhcN* | orf, hypothetical protein [b3238] | -14.739 | 0.020 |
| *pepE* | peptidase E, a dipeptidase where amino-terminal residue is aspartate [b4021] | 2.207 | 0.005 |
| *menE* | o-succinylbenzoate-CoA ligase [b2260] | -7.175 | 0.006 |
| *ompW* | putative outer membrane protein [b1256] | 2.057 | 0.036 |
| *otsA* | trehalose-6-phosphate synthase [b1896] | -2.321 | 0.007 |
| *vacJ* | VacJ lipoprotein precursor [c_2890] | -2.057 | 0.004 |
| *ECs3728* | hypothetical protein [ECs3728] | -14.230 | 0.013 |
| *yjiX* | orf, hypothetical protein [b4353] | -2.749 | 0.009 |
| *ECs0257* | flagellar biosynthesis [ECs0257] | -2.075 | 0.032 |
| *yedE* | putative transport system permease protein [b1929] | 2.633 | 0.004 |
| *cysN* | ATP-sulfurylase [b2751] | -2.260 | 0.024 |
| *yrbL* | orf, hypothetical protein [b3207] | -14.074 | 0.000 |
| *yeiN* | orf, hypothetical protein [b2165] | 8.602 | 0.003 |
|  | orf, hypothetical protein [b0302] | -4.134 | 0.000 |
| *ECs1836* | anthranilate synthase component I [ECs1836] | 2.965 | 0.045 |
| *yiiR* | orf, hypothetical protein [b3921] | 2.035 | 0.028 |
| *insE* | putative factor [b0298] | -2.098 | 0.049 |
| *yajI* | orf, hypothetical protein [b0412] | 2.906 | 0.021 |
| *alsK* | putative NAGC-like transcriptional regulator [b4084] | 2.179 | 0.015 |
| *modA* | Molybdate-binding periplasmic protein precursor [c_0840] | -2.283 | 0.006 |
|  | Conserved hypothetical protein [c_2522] | -20.682 | 0.004 |
| *cydB* | cytochrome d terminal oxidase polypeptide subunit II [b0734] | 2.185 | 0.026 |
| *ECs3810* | transketolase 1 isozyme [ECs3810] | 2.196 | 0.043 |
| *rcsF* | Protein rcsF [c_0237] | 2.401 | 0.009 |
| *slmA* | putative transcriptional regulator [b3641] | 2.000 | 0.016 |
| *vacJ* | lipoprotein precursor [b2346] | -2.148 | 0.011 |
| *osmY* | hyperosmotically inducible periplasmic protein [b4376] | -2.274 | 0.038 |
| *yedE* | putative transport system permease protein [Z3019] | 2.898 | 0.001 |
| *mepA* | murein DD-endopeptidase, penicillin-insensitive [b2328] | -3.059 | 0.021 |
| *yfcB* | putative adenine-specific methylase [Z3593] | 2.282 | 0.010 |
| *tdcD* | putative kinase [Z4467] | 6.463 | 0.007 |
| *ybhN* | orf, hypothetical protein [Z1007] | -2.393 | 0.050 |
|  | Hypothetical protein [c_1210] | -9.871 | 0.014 |
|  | Hypothetical protein [c_5458] | -2.732 | 0.026 |
| *acnA* | aconitate hydrase 1 [b1276] | -3.041 | 0.032 |
|  | Putative conserved protein [c_3873] | 6.483 | 0.012 |
| *mhpC* | 2-hydroxy-6-ketonona-2,4-dienedioic acid hydrolase [b0349] | 2.410 | 0.006 |
|  | orf, hypothetical protein [Z3657] | -3.536 | 0.005 |
| *rplQ* | 50S ribosomal subunit protein L17 [b3294] | 2.006 | 0.039 |
| *Z3275* | orf Unknown function [Z3275] | 2.869 | 0.004 |
| *ydeB* | orf, hypothetical protein [Z2172] | -4.653 | 0.001 |
| *lacZ* | beta-D-galactosidase [b0344] | -2.129 | 0.001 |
| *trxB* | Thioredoxin reductase [c_1025] | 2.893 | 0.039 |
| *yebS* | orf, hypothetical protein [b1833] | -2.981 | 0.009 |
| *ygaW* | orf, hypothetical protein [b2670] | 3.067 | 0.045 |
| *fimB* | recombinase involved in phase variation; regulator for fimA [b4312] | 2.015 | 0.038 |
| *ypeC* | orf, hypothetical protein [b2390] | -3.631 | 0.003 |
| *envR* | putative transcriptional regulator [b3264] | -2.428 | 0.000 |
| *yhaJ* | putative transcriptional regulator LYSR-type [b3105] | 2.058 | 0.005 |
|  | Hypothetical protein [c_4115] | 2.171 | 0.044 |
| *mtlR* | repressor for mtl [b3601] | -7.859 | 0.001 |
|  | anthranilate synthase component I [Z2547] | 4.645 | 0.014 |
|  | Hypothetical protein [c_3270] | -8.527 | 0.008 |
| *sgaE* | putative epimerase/aldolase [ECs5174] | 2.083 | 0.002 |
| *ECs1129* | hydrogenase-1 large subunit [ECs1129] | -10.628 | 0.000 |
| *L7085* | hypothetical protein [L7085] | -2.813 | 0.007 |
| *inaA* | pH-inducible protein involved in stress response [b2237] | -9.549 | 0.006 |
| *uxaB* | altronate oxidoreductase [b1521] | -3.578 | 0.019 |
|  | putative L-serine dehydratase [b3111] | 5.960 | 0.006 |
| *rpmA* | 50S ribosomal subunit protein L27 [b3185] | 2.015 | 0.038 |
| *ECs2136* | hypothetical protein [ECs2136] | -3.605 | 0.040 |
| *rsxG* | orf, hypothetical protein [b1631] | 2.379 | 0.028 |
| *xisW* | putative excisionase for bacteriophage BP-933W [Z1425] | -11.808 | 0.009 |
| *yadP* | 2-5 RNA ligase [c_0180] | 2.122 | 0.017 |
| *artM* | arginine 3rd transport system permease protein [b0861] | -2.182 | 0.025 |
| *acrB* | acridine efflux pump [b0462] | -3.316 | 0.022 |
| *maa* | putative transferase [b0459] | 2.707 | 0.014 |
| *fepD* | Ferric enterobactin transport system permease protein fepD [c_0677] | 5.634 | 0.017 |
| *yajG* | putative polymerase/proteinase [b0434] | 2.025 | 0.030 |
| *ychM* | orf, hypothetical protein [Z1977] | 3.760 | 0.001 |
| *idi* | putative enzyme [b2889] | -2.802 | 0.001 |
| *aceA* | Isocitrate lyase [c_4972] | 3.121 | 0.038 |
| *poxB* | pyruvate oxidase [Z1105] | -2.917 | 0.006 |
| *mchC* | MchC protein [c_1229] | -24.020 | 0.031 |
| *hupA* | DNA-binding protein HU-alpha [b4000] | 2.567 | 0.000 |
| *trxB* | thioredoxin reductase [b0888] | 3.320 | 0.011 |
| *allS* | putative transcriptional regulator LYSR-type [b0504] | 4.413 | 0.008 |
| *ybbQ* | 2-hydroxy-3-oxopropionate reductase [c_0624] | 7.510 | 0.018 |
| *fecA* | outer membrane receptor; citrate-dependent iron transport, outer membrane receptor [b4291] | -2.214 | 0.003 |
| *ymgC* | orf, hypothetical protein [b1167] | -2.773 | 0.009 |
| *ydaU* | orf, hypothetical protein [b1359] | -17.056 | 0.017 |
| *ymgI* | hypothetical protein [b4593] | -16.020 | 0.023 |
| *yceA* | orf, hypothetical protein [b1055] | 2.014 | 0.019 |
|  | FlRd-NAD [c_3267] | 3.143 | 0.011 |
| *ybjC* | orf, hypothetical protein [b0850] | -8.726 | 0.005 |
| *purH* | phosphoribosylaminoimidazolecarboxamideformyltra nsferase [ECs4929] | 2.093 | 0.005 |
|  | Hypothetical protein [c_3397] | -18.781 | 0.001 |
| *crcA* | CrcA protein [c_0713] | -17.892 | 0.005 |
| *prpB* | putative phosphonomutase 2 [b0331] | -3.720 | 0.025 |
| *guaC* | GMP reductase [b0104] | 3.007 | 0.004 |
| *trpD* | anthranilate synthase component II, glutamine amidotransferase and phosphoribosylanthranilate transferase [b1263] | 5.043 | 0.032 |
| *fepC* | ATP-binding component of ferric enterobactin transport [b0588] | 2.053 | 0.015 |
| *yohN* | orf, hypothetical protein [b2107] | 2.562 | 0.015 |
| *ompC* | outer membrane protein 1b [Z3473] | 2.068 | 0.003 |
| *hyi* | glyoxylate-induced protein [b0508] | 9.526 | 0.034 |
| *rcsF* | regulator in colanic acid synthesis interacts with [Z0208] | 2.497 | 0.004 |
| *tktA* | transketolase 1 isozyme [Z4279] | 2.310 | 0.035 |
| *yeeD* | orf, hypothetical protein [b2012] | -2.984 | 0.035 |
| *tdcB* | threonine dehydratase, catabolic [b3117] | 4.718 | 0.016 |
| *ybjG* | orf, hypothetical protein [b0841] | 2.541 | 0.004 |
| *Z3924* | partial putative transposase [Z3924] | -24.283 | 0.011 |
| *prpD* | orf, hypothetical protein [Z0429] | -3.389 | 0.025 |
| *ykgG* | orf Unknown function [Z0386] | 2.748 | 0.017 |
| *dusA* | orf, hypothetical protein [b4049] | 2.317 | 0.006 |
| *ECs4307* | hypothetical protein [ECs4307] | -5.374 | 0.012 |
|  | D-alanine--D-alanine ligase A [c_0487] | 2.049 | 0.033 |
| *atpA* | membrane-bound ATP synthase, F1 sector, alpha-subunit [b3734] | 2.457 | 0.019 |
| *ECs3631* | putative transport protein [ECs3631] | 2.146 | 0.022 |
| *ECs1833* | tryptophan synthase beta protein [ECs1833] | 2.463 | 0.049 |
| *mdtG* | putative transport protein [b1053] | -2.947 | 0.012 |
|  | yapH homolog [c_2895] | -8.437 | 0.007 |
| *yrbD* | orf, hypothetical protein [b3193] | -2.073 | 0.019 |
| *cbrC* | orf, hypothetical protein [b3717] | 2.016 | 0.018 |
|  | anthranilate synthase component I [Z2546] | 3.211 | 0.013 |
|  | Hypothetical transporter ydgR [c_2026] | 3.792 | 0.015 |
| *prs* | phosphoribosylpyrophosphate synthetase [b1207] | 2.613 | 0.014 |
| *ydhS* | orf, hypothetical protein [b1668] | -2.073 | 0.012 |
| *lacA* | thiogalactoside acetyltransferase [b0342] | -2.523 | 0.020 |
| *yohN* | Hypothetical protein yohN precursor [c_2634] | 2.756 | 0.013 |
| *insF* | IS3 putative transposase [b0299] | -2.331 | 0.044 |
| *ulaA* | orf, hypothetical protein [b4193] | 2.655 | 0.020 |
| *yibT* | predicted protein [b4554] | -12.996 | 0.001 |
| *ykgF* | orf, hypothetical protein [b0307] | 2.447 | 0.030 |
| *yfeN* | putative sugar hydrolase [b2408] | -2.145 | 0.014 |
| *rplU* | 50S ribosomal subunit protein L21 [b3186] | 2.276 | 0.019 |
| *pldA* | outer membrane phospholipase A [b3821] | 2.467 | 0.048 |
| *tuf* | protein chain elongation factor EF-Tu [ECs4903] | 2.029 | 0.008 |
| *yheF* | Probable general secretion pathway protein D precursor [c_4096] | -4.418 | 0.020 |
| *ubiX* | 3-octaprenyl-4-hydroxybenzoate carboxy-lyase [b2311] | 2.238 | 0.023 |
| *yejO* | putative ATP-binding component of a transport system [b2190] | -2.260 | 0.025 |
| *gcl* | glyoxylate carboligase [b0507] | 10.599 | 0.031 |
| *yhbW* | Hypothetical protein yhbW [c_3913] | -4.100 | 0.010 |
| *glcF* | glycolate oxidase iron-sulfur subunit [b2978] | 22.604 | 0.001 |
| *rnt* | RNase T, degrades tRNA [b1652] | 2.347 | 0.014 |
|  | Putative superinfection exclusion protein B of prophage [c_1543] | -4.974 | 0.018 |
| *ppdA* | prepilin peptidase dependent protein A [b2826] | 3.641 | 0.047 |
| *ompW* | Outer membrane protein W precursor [c_1722] | 2.287 | 0.019 |
| *yhaR* | TdcF protein [c_3871] | 8.562 | 0.004 |
|  | PTS system, mannose-specific IID component [c_4982] | -2.913 | 0.010 |
| *tppB* | putative transport protein [b1634] | 3.400 | 0.004 |
| *nfsA* | modulator of drug activity A [b0851] | -6.059 | 0.007 |
| *yhbW* | putative enzyme [b3160] | -3.578 | 0.009 |
| *yfgO* | putative permease [b2493] | -2.001 | 0.048 |
| *ygaD* | orf, hypothetical protein [b2700] | 3.528 | 0.001 |
| *tsf* | protein chain elongation factor EF-Ts [b0170] | 2.129 | 0.035 |
| *uraA* | uracil transport [b2497] | 2.151 | 0.016 |
|  | 2,5-diketo-D-gluconic acid reductase A [c_0417] | -2.972 | 0.010 |
| *yncE* | putative receptor [b1452] | 2.078 | 0.001 |
| *fucA* | L-fuculose-1-phosphate aldolase [b2800] | 2.066 | 0.001 |
| *adiY* | putative ARAC-type regulatory protein [b4116] | 6.517 | 0.027 |
| *argO* | orf, hypothetical protein [b2923] | 2.632 | 0.016 |
| *yeiC* | putative kinase [b2166] | 6.440 | 0.007 |
| *panB* | 3-methyl-2-oxobutanoate hydroxymethyltransferase [b0134] | -3.933 | 0.007 |
| *ymgG* | orf, hypothetical protein [b1172] | 4.471 | 0.021 |
| *glcD* | glycolate oxidase subunit D [b2979] | 12.784 | 0.001 |
| *ECs2913* | hypothetical protein [ECs2913] | 2.904 | 0.012 |
| *glcF* | glycolate oxidase iron-sulfur subunit [b2978] | 9.910 | 0.007 |
| *glcG* | orf, hypothetical protein [b2977] | 12.756 | 0.013 |
| *panB* | 3-methyl-2-oxobutanoate hydroxymethyltransferase [c_0165] | -4.259 | 0.004 |
| *cysS* | cysteine tRNA synthetase [b0526] | 2.615 | 0.003 |
| *Z0665* | putative transport Not classified [Z0665] | 19.887 | 0.010 |
| *glgS* | glycogen biosynthesis, rpoS dependent [b3049] | -20.250 | 0.006 |
| *dnaG* | DNA biosynthesis; DNA primase [b3066] | -2.395 | 0.007 |
| *yaiY* | orf, hypothetical protein [b0379] | 2.369 | 0.038 |
| *xdhC* | putative dehydrogenase [b2868] | 2.289 | 0.011 |
| *phnG* | PhnG protein [c_5107] | -2.597 | 0.003 |
| *rpiR* | transcriptional repressor of rpiB expression [b4089] | 2.471 | 0.012 |
| *dgoT* | D-galactonate transport [b3691] | 2.508 | 0.005 |
| *marC* | orf, hypothetical protein [b1529] | -4.099 | 0.006 |
| *aslB* | Putative arylsulfatase Regulatory protein [c_4718] | -3.476 | 0.010 |
| *alsB* | putative LACI-type transcriptional regulator [b4088] | 2.830 | 0.000 |
| *yedP* | orf, hypothetical protein [b1955] | -2.130 | 0.031 |
| *ECs4086* | hypothetical protein [ECs4086] | -12.914 | 0.000 |
| *hybA* | hydrogenase-2 small subunit [b2996] | -2.168 | 0.033 |
| *insB* | IS1 protein InsB [b0988] | -2.085 | 0.046 |
| *gloB* | probable hydroxyacylglutathione hydrolase [b0212] | -2.080 | 0.016 |
| *ycfJ* | orf, hypothetical protein [b1110] | 3.167 | 0.014 |
| *yfcB* | Hypothetical adenine-specific methylase yfcB [c_2876] | 2.241 | 0.025 |
|  | predicted protein | -2.929 | 0.029 |
|  | Hypothetical protein [c_2740] | 2.925 | 0.005 |
| *ybaO* | putative LRP-like transcriptional regulator [b0447] | -2.050 | 0.013 |
| *prpD* | hypothetical protein [ECs0387] | -3.145 | 0.024 |
| *pyrC* | dihydro-orotase [b1062] | 2.667 | 0.029 |
| *glcB* | malate synthase G [b2976] | 7.675 | 0.011 |
| *vacJ* | lipoprotein precursor [Z3610] | -2.170 | 0.010 |
| *lacA* | thiogalactoside acetyltransferase [ECs0395] | -2.784 | 0.002 |
|  | Prophage Qin DNA packaging protein NU1 homolog [c_1444] | -3.194 | 0.014 |
| *aroH* | 3-deoxy-D-arabinoheptulosonate-7-phosphate synthase [b1704] | 2.023 | 0.013 |
| *ymgA* | orf, hypothetical protein [b1165] | -3.997 | 0.037 |
| *rpmD* | 50S ribosomal subunit protein L30 [b3302] | 2.044 | 0.048 |
| *aceB* | malate synthase A [b4014] | 3.023 | 0.020 |
| *fhlA* | formate hydrogen-lyase transcriptional activator for fdhF, hyc and hyp operons [b2731] | -2.092 | 0.002 |
| *yedE* | Hypothetical protein yedE [c_2344] | 2.601 | 0.001 |
| *ybaL* | putative transport protein [b0478] | -2.276 | 0.003 |
| *purH* | phosphoribosylaminoimidazolecarboxamide formyltransferase = AICAR formyltransferase IMP cyclohydrolase [Z5583] | 2.622 | 0.035 |
| *yicG* | orf, hypothetical protein [b3646] | 2.114 | 0.023 |
| *yeeR* | orf, hypothetical protein [b2001] | 2.020 | 0.015 |
|  | IS186 and IS421 hypothetical protein [b0017] | 2.264 | 0.001 |
| *aslB* | putative arylsulfatase regulator [b3800] | -5.000 | 0.002 |
| *yjiY* | putative carbon starvation protein [b4354] | -4.235 | 0.011 |
| *lacA* | thiogalactoside acetyltransferase [Z0438] | -2.566 | 0.013 |
| *yrbE* | orf, hypothetical protein [b3194] | -2.567 | 0.003 |
| *trpE* | anthranilate synthase component I [b1264] | 3.149 | 0.014 |
| *tdcC* | anaerobically inducible L-threonine, L-serine permease [b3116] | 4.341 | 0.010 |
|  | Hypothetical protein [c_1718] | 4.491 | 0.014 |
| *pps* | phosphoenolpyruvate synthase [b1702] | 2.119 | 0.019 |
| *ykgG* | putative transporter [b0308] | 2.529 | 0.009 |
| *wzzB* | regulator of length of O-antigen component of [Z3189] | 2.819 | 0.029 |
| *yrbF* | putative ATP-binding component of a transport system [b3195] | -3.021 | 0.000 |
| *tdcE* | probable formate acetyltransferase 3 [b3114] | 6.809 | 0.008 |
| *tgt* | tRNA-guanine transglycosylase [b0406] | 2.032 | 0.028 |
| *tktA* | transketolase 1 isozyme [b2935] | 2.257 | 0.044 |
| *frwC* | PTS system, fructose-like enzyme II component [b3949] | 2.199 | 0.035 |
|  | Hypothetical protein [c_4650] | 2.687 | 0.037 |
| *phoU* | negative regulator for pho regulon and putative enzyme in phosphate metabolism [b3724] | 2.057 | 0.044 |
| *acrA* | acridine efflux pump [b0463] | 0.408 | 0.007 |
| *ykgE* | putative dehydrogenase subunit [b0306] | 3.037 | 0.033 |
| *menC* | o-succinylbenzoyl-CoA synthase; conversion of chorismate to 2-o-succinylbenzoyl-CoA [b2261] | -9.097 | 0.001 |
| *yeiM* | putative transport system permease protein [b2164] | 4.911 | 0.037 |
| *pdxY* | pyridoxal kinase 2 / pyridoxine kinase [b1636] | 2.390 | 0.009 |
| *rnd* | RNase D, processes tRNA precursor [b1804] | 2.144 | 0.038 |
| *Z5852* | orf Unknown function [Z5852] | -2.585 | 0.027 |
| *pstS* | high-affinity phosphate-specific transport system; periplasmic phosphate-binding protein [b3728] | 2.570 | 0.027 |
| *glcB* | Malate synthase G [c_3705] | 7.863 | 0.024 |
| *rsmC* | putative enzyme [b4371] | 2.526 | 0.035 |
| *insL* | IS186 hypothetical protein [b2394] | 3.017 | 0.003 |
| *yeaZ* | orf, hypothetical protein [b1807] | 2.972 | 0.015 |
| *Z5694* | orf Unknown function [Z5694] | 3.456 | 0.003 |
| *ebgA* | evolved beta-D-galactosidase, alpha subunit; cryptic gene [b3076] | 2.527 | 0.032 |
| *xylR* | Xylose operon Regulatory protein [c_4389] | 4.818 | 0.012 |
| *queA* | synthesis of queuine in tRNA; probably S-adenosylmethionine:tRNA ribosyltransferase-isomerase [b0405] | 2.047 | 0.040 |
| *nupC* | permease of transport system for 3 nucleosides [b2393] | 2.834 | 0.013 |
| *ulaF* | putative epimerase/aldolase [b4198] | 2.567 | 0.041 |
|  | fertility inhibition protein | -3.669 | 0.022 |
| *yneJ* | putative transcriptional regulator LYSR-type [Z2177] | 2.721 | 0.003 |
| *yohN* | orf, hypothetical protein [b2107] | 3.230 | 0.001 |
| *ybgQ* | putative outer membrane protein [b0718] | 5.536 | 0.029 |
| *ECs5173* | putative hexulose-6-phosphate isomerase [ECs5173] | 2.643 | 0.031 |
| *mutM* | formamidopyrimidine DNA glycosylase [b3635] | 2.426 | 0.045 |
