# Supplementary material for: DNA microarray of global transcription factor mutant reveals membrane-related proteins involved in n-butanol tolerance in Escherichia coli
Source: Biotechnol Biofuels. 2016 Jun 1;9:114. doi: 10.1186/s13068-016-0527-9 (PMC4888631; doi:10.1186/s13068-016-0527-9)
Supplement: Supplementary file 4 — 10.1186/s13068-016-0527-9 Transcription profile analysis of genes exhibiting significantly different expression levels in σ70 mutant B8 and WT. Hierarchical clustering using differentially expressed genes (probe sets) (p < 0.05; FC ≥ 2). Abscissa represents samples of B8 and WT, the ordinate represents different genes. Three biological replicates were performed. [file 13068_2016_527_MOESM4_ESM.docx]

**DNA Microarray of Global Transcription Factor Mutant Reveals Membrane-Related Proteins Involved in n-Butanol Tolerance in *Escherichia coli***

# Supplementary Online Material

**Additional file 4**. Transcription profile analysis of genes exhibiting significantly different expression levels in σ^70^ mutant B8 and WT. Hierarchical clustering using differentially expressed genes (probe sets) (*p*<0.05; FC≥2). Abscissa represents samples of B8 and WT, the ordinate represents different genes. Three biological replicates were performed. (Fig. S3)


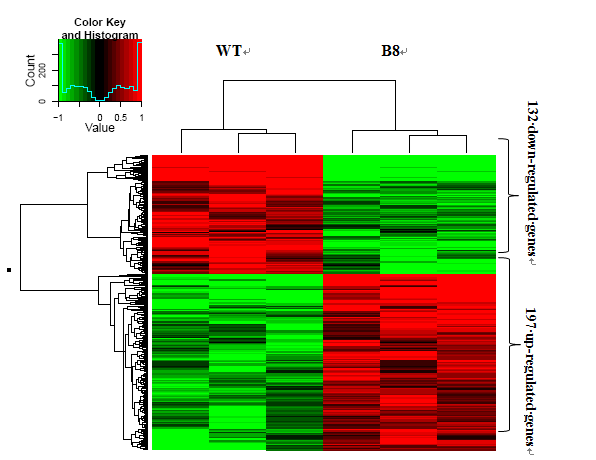
**Fig. S3**
