# Supplementary material for: DNA microarray of global transcription factor mutant reveals membrane-related proteins involved in n-butanol tolerance in Escherichia coli
Source: Biotechnol Biofuels. 2016 Jun 1;9:114. doi: 10.1186/s13068-016-0527-9 (PMC4888631; doi:10.1186/s13068-016-0527-9)
Supplement: Supplementary file 5 — 10.1186/s13068-016-0527-9 Analysis of surface hydrophobicity of E. coli knockout strains △yghW, △yibT and JM109 (control) by MATS. Three biological replicates were performed. [file 13068_2016_527_MOESM5_ESM.docx]

**DNA Microarray of Global Transcription Factor Mutant Reveals Membrane-Related Proteins Involved in n-Butanol Tolerance in *Escherichia coli***

# Supplementary Online Material

**Additional file 5.** Analysis of surface hydrophobicity of *E. coli* knockout strains △*yghW*, △*yibT* and JM109 (control) by MATS. Three biological replicates were performed. (Fig. S4)

**Fig. S4**
