# Supplementary material for: DNA microarray of global transcription factor mutant reveals membrane-related proteins involved in n-butanol tolerance in Escherichia coli
Source: Biotechnol Biofuels. 2016 Jun 1;9:114. doi: 10.1186/s13068-016-0527-9 (PMC4888631; doi:10.1186/s13068-016-0527-9)
Supplement: Supplementary file 6 — 10.1186/s13068-016-0527-9 Contact angle measurement of E. coli knockout strains (A) JM109 (control), (B) △yghW, and (C) △yibT. Three biological replicates were performed. [file 13068_2016_527_MOESM6_ESM.docx]

**DNA Microarray of Global Transcription Factor Mutant Reveals Membrane-Related Proteins Involved in n-Butanol Tolerance in *Escherichia coli***

# Supplementary Online Material

**Additional file 6**. Contact angle measurement of *E. coli* knockout strains (A) JM109 (control), (B) △*yghW*, and (C) △*yibT.* Three biological replicates were performed. (Fig. S5)

**Fig. S5**
