# Supplementary material for: DNA microarray of global transcription factor mutant reveals membrane-related proteins involved in n-butanol tolerance in Escherichia coli
Source: Biotechnol Biofuels. 2016 Jun 1;9:114. doi: 10.1186/s13068-016-0527-9 (PMC4888631; doi:10.1186/s13068-016-0527-9)
Supplement: Supplementary file 7 — 10.1186/s13068-016-0527-9 Analysis of n-butanol tolerance of various gene overexpression strains with or without 0.8 % n-butanol. Strains were pre-cultured at 37 °C with addition of 0.2 mM IPTG at 0.3 OD660. Then 0.8 % (v/v) butanol was added at 0.8 OD660 for further incubation at 30 °C for 8 h. Three biological replicates were performed. [file 13068_2016_527_MOESM7_ESM.docx]

**DNA Microarray of Global Transcription Factor Mutant Reveals Membrane-Related Proteins Involved in n-Butanol Tolerance in *Escherichia coli***

# Supplementary Online Material

**Additional file 7**. Analysis of n-butanol tolerance of various gene overexpression strains with or without 0.8% n-butanol. Strains were pre-cultured at 37°C with addition of 0.2 mM IPTG at 0.3 OD_660_. Then 0.8% (v/v) butanol was added at 0.8 OD_660_ for further incubation at 30°C for 8 h. Three biological replicates were performed. (Fig. S6)

**Fig. S6**
