# Supplementary material for: DNA microarray of global transcription factor mutant reveals membrane-related proteins involved in n-butanol tolerance in Escherichia coli
Source: Biotechnol Biofuels. 2016 Jun 1;9:114. doi: 10.1186/s13068-016-0527-9 (PMC4888631; doi:10.1186/s13068-016-0527-9)
Supplement: Supplementary file 8 — 10.1186/s13068-016-0527-9 Pathways of glycolate metabolism and tricarboxylic acid cycle in Escherichia coli. [file 13068_2016_527_MOESM8_ESM.docx]

**DNA Microarray of Global Transcription Factor Mutant Reveals Membrane-Related Proteins Involved in n-Butanol Tolerance in *Escherichia coli***

# Supplementary Online Material

**Additional file 8**. Pathways of glycolate metabolism and tricarboxylic acid cycle in *Escherichia coli* (Fig*.* S7)


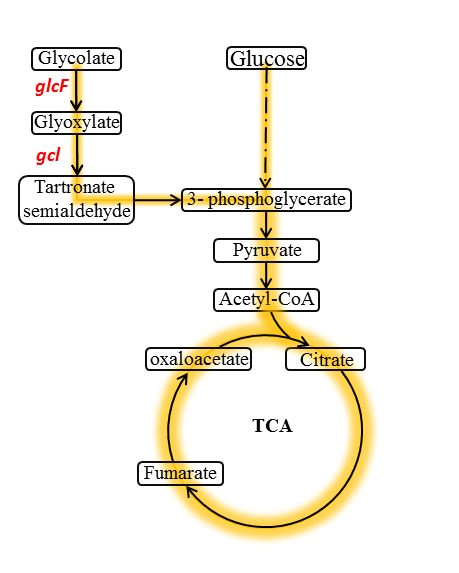


**Fig. S7**
