# Supplementary material for: DNA microarray of global transcription factor mutant reveals membrane-related proteins involved in n-butanol tolerance in Escherichia coli
Source: Biotechnol Biofuels. 2016 Jun 1;9:114. doi: 10.1186/s13068-016-0527-9 (PMC4888631; doi:10.1186/s13068-016-0527-9)
Supplement: Supplementary file 9 — 10.1186/s13068-016-0527-9 Pyruvate and fumarate levels in cell cultures of gcl and glcF overexpression strains with or without butanol. [file 13068_2016_527_MOESM9_ESM.docx]

**DNA Microarray of Global Transcription Factor Mutant Reveals Membrane-Related Proteins Involved in n-Butanol Tolerance in *Escherichia coli***

# Supplementary Online Material

**Additional file 9.** Pyruvate and fumarate levels in cell cultures of *gcl* and *glcF* overexpression strains with or without butanol (Table S4).

**Table S4** Pyruvate and fumarate levels in cell cultures of *gcl* and *glcF* overexpression strains with or without butanol.

|  | Pyruvate (mg/L) | |  | Fumarate (g/L) | |
| --- | --- | --- | --- | --- | --- |
| Strain | Without butanol | With 0.8%(v/v) butanol |  | Without butanol | With 0.8%(v/v) butanol |
| Control | 0.107±0.006 | 0.185±0.015 |  | 0.047±0.012 | 0.082±0.002 |
| pQE-*gcl* | 0.499±0.164 | 0.745±0.022 |  | 0.080±0.009 | 0.077±0.006 |
| pQE-*glcF* | 0.174±0.006 | 0.276±0.016 |  | 0.051±0.006 | 0.074±0.008 |

Strains were cultured in LBG medium with or without 0.8% (v/v) butanol to stationary phase. Cell culture was centrifuged and the supernatant was analyzed by HPLC. *E. coli* carrying empty plasmid pQE-80L was used as control. Three biological replicates were performed.
