# Supplementary material for: DNA microarray of global transcription factor mutant reveals membrane-related proteins involved in n-butanol tolerance in Escherichia coli
Source: Biotechnol Biofuels. 2016 Jun 1;9:114. doi: 10.1186/s13068-016-0527-9 (PMC4888631; doi:10.1186/s13068-016-0527-9)
Supplement: Supplementary file 10 — 10.1186/s13068-016-0527-9 Strains and plasmids used in this study. [file 13068_2016_527_MOESM10_ESM.docx]

**DNA Microarray of Global Transcription Factor Mutant Reveals Membrane-Related Proteins Involved in n-Butanol Tolerance in *Escherichia coli***

# Supplementary Online Material

**Additional file 10.** Strains and plasmids used in this study (Table S1).

**Table S1** Strains and plasmids used in this study

| Strains and plasmids | Description | Reference |
| --- | --- | --- |
| JM109 | F’(traD36, proAB+, lacIq, Δ(lacZ)M15) endA1 recA1 hsdR17 (r− K , m+ K) mcrA supE44 λ− gyrA96 relA1 Δ(lac-proAB) thi−1 | Takara |
| JM109(Δ*yghW*) | Same as JM109, but with Δ*yghW* | This study |
| JM109(Δ*yibT*) | Same as JM109, but with Δ*yibT* | This study |
| JM109(Δ*ymgI*) | Same as JM109, but with Δ*ymgI* | This study |
| JM109(Δ*yhcN*) | Same as JM109, but with Δ*yhcN* | This study |
| JM109(Δ*yrbL*) | Same as JM109, but with Δ*yrbL* | This study |
| JM109(Δ*Ecs4086*) | Same as JM109, but with Δ *Ecs4086* | This study |
| JM109(pQE) | Same as JM109, but with plasmid pQE | This study |
| JM109(*glcF*-pQE) | Same as JM109, but with *glcF*-pQE | This study |
| JM109(*glcA*-pQE) | Same as JM109, but with *glcA*-pQE | This study |
| JM109(*glcD*-pQE)  JM109(*gcl*-pQE)  JM109(*glcG*-pQE) | Same as JM109, but with *glcD*-pQE  Same as JM109, but with *gcl*-pQE  Same as JM109, but with *glcG*-pQE | This study  This study  This study |
| pKD13, pKD46, pCP20 | Gene disruption set | Datsenko & Wanner |
| pQE80L | Expression vector | Qiagen |
