# Supplementary material for: DNA microarray of global transcription factor mutant reveals membrane-related proteins involved in n-butanol tolerance in Escherichia coli
Source: Biotechnol Biofuels. 2016 Jun 1;9:114. doi: 10.1186/s13068-016-0527-9 (PMC4888631; doi:10.1186/s13068-016-0527-9)
Supplement: Supplementary file 11 — 10.1186/s13068-016-0527-9 Primes used in this study. [file 13068_2016_527_MOESM11_ESM.docx]

**DNA Microarray of Global Transcription Factor Mutant Reveals Membrane-Related Proteins Involved in n-Butanol Tolerance in *Escherichia coli***

# Supplementary Online Material

**Additional file 11.** Primes used in this study (Table S2).

**Table S2** Primes used in this study

| Purpose | Prime | Sequence (5'→3') |
| --- | --- | --- |
| Amplification | *rpoD*-F | 5'-AACCTAGGAGCTCTGATTTAACGGCTTAAGTGCCGAAGAGC-3' |
| of *rpoD* | *rpoD*-R | 5'-TGGAAGCTTTAACGCCTGATCCGGCCTACCGATTA-3' |
| Gene knockout | Δ*yibT*-F | AAAATTGGCGGCCGCGCATTCCCGGCGCGGGGTAATACGGAGATATCATCGTGTAGGCTGGAGCTGCTTC |
|  | Δ*yibT*-R | ATTTCCAAAATCAGCTTAAAAAAAGCCTTTCAAAAGTAAGCAACGTCTGCATTCCGGGGATCCGTCGACC |
|  | Δ*yghW*-F | TCAAACATACGTATTATCTTGCTTTAATTAATTACACTAATGCTTCTTCCGTGTAGGCTGGAGCTGCTTC |
|  | Δ*yghW*-R | CGTTCACCTCATTCGCTATATATTGTCATATATAGCGATATTTTAGGCAAATTCCGGGGATCCGTCGACC |
|  | Δ*ymgI*-F | CATTGCGTTAACAGTCAATGACACTTCTTGAATAAAAATATTGTCAATCAGTGTAGGCTGGAGCTGCTTC |
|  | Δ*ymgI*-R | AGCAGAACAATGCTGAGATAAGCCCGAAGGCAAGTATTTTTTTCTTCAACATTCCGGGGATCCGTCGACC |
|  | Δ*yhcN*-F | TGGGTCACGAAACAAAGGCCCAGCTAAAAGATTATGTCGAGGTAAAAATCGTGTAGGCTGGAGCTGCTTC |
|  | Δ*yhcN*-R | AAAAAAGCCCCCGAACCGGGGGCAATATCGTCGGACAAGACGATGAGGGTATTCCGGGGATCCGTCGACC |
|  | Δ*yrbL*-F | AGTTAATCGACCATACTGGAGATCGTCAGAAAATATTTCCAGGAGATGGCGTGTAGGCTGGAGCTGCTTC |
|  | Δ*yrbL*-R | CAGATGTATCAGTTAGGCGGTGAGCCGTTTATGCAACAACACCAGCTGGAATTCCGGGGATCCGTCGACC |
|  | Δ*ECs4086-*F | AGTTAATCGACCATACTGGAGATCGTCAGAAAATATTTCCAGGAGATGGCGTGTAGGCTGGAGCTGCTTC |
|  | Δ*ECs4086*-R | CAGATGTATCAGTTAGGCGGTGAGCCGTTTATGCAACAACACCAGCTGGAATTCCGGGGATCCGTCGACC |
| Confirmation of knockout | C-*yibT*-F | GTCATCTTTACCGGGAACCTAAACT |
|  | C-*yibT*-R | AATGGTCCTTTCACTGACTGAGCTG |
|  | C-*yghW*-F | CTTCGTTTTAGCGCCCCGCCGCAGT |
|  | C-*yghW*-R | AGAACGACTTTTGCGCACGCTATTT |
|  | C-*ymgI*-F | AAATCAGATTATCTTACCTTTACAA |
|  | C-*ymgI*-R | TTCATATCCGCCATCGCTGGCGTGG |
|  | C-*yhcN*-F | TTAGTGTATACTTGATTTTGTGATA |
|  | C-*yhcN*-R | GTTTTAATGTTCGTCATTAGCG |
|  | C-*yrbL*-F | AGAGGCATTGTTTAGGTTTTGTTTA |
|  | C-*yrbL*-R | TAGCCGTCAGGCGTGGATTTTA |
|  | C-*ECs4086-*F | AGAAGCATTGTTTAGGTTTCGTTTA |
|  | C-*ECs4086*-R | CAGCCGTCAGGCGTGGATTTTACGG |
|  | K_2_ | CGGTGCCCTGAATGAACTGC |
|  | K_t_ | CGGCCACAGTCGATGAATCC |
|  |  |  |
| Gene Overexpression | *glcF*-F | CGGGATCCATGCAAACCCAATTAACTGA(*BamH* I) |
|  | *glcF*-R | CCCAAGCTTTTATTCCTTTTCAAGGGCTT (*Hind* III) |
|  | *glcA*-F | CGGGATCCATGGTTACCTGGACCCAAAT (*BamH* I) |
|  | *glcA*-R | CGAGCTCGTTACGAGACTAACATCCCGG (*Sac* I) |
|  | *gcl*-F | CGGGATCCATGGCAAAAATGAGAGCCGT (*BamH* I) |
|  | *gcl*-R | CCCAAGCTTTTATTCATAGTGCATGAAGC (*Hind* III) |
|  | *glcD*-F | CGGATCCATGAGCATCTTGTACGAAGAGCG(*BamH* I) |
|  | *glcD*-R | CCCAAGCTTTCAGAAACGCTCCAGTTCAGG(*Hind* III) |
|  | *glcG*-F | CGGGATCCATGAAAACTAAAGTCATTCTTAGCCAGC(*BamH* I) |
|  | *glcG*-R | CCCAAGCTTTTATTTCGCCAACACCGCTG(*Hind* III) |
|  | pQE-F | TGAGCGGATAACAAT TTCAC |
|  | pQE-R | GTTCTGAGGTCATTACTGG |
